# Supplementary material for: Identified Cellular Correlates of Neocortical Ripple and High-Gamma Oscillations during Spindles of Natural Sleep
Source: Neuron. 2016 Nov 23;92(4):916–28. doi: 10.1016/j.neuron.2016.09.032 (PMC5130902; doi:10.1016/j.neuron.2016.09.032)
Supplement: Document S1. Figures S1–S5 and Table S1 [file mmc1.pdf]

Neuron, Volume *92*

Supplemental Information

Identified Cellular Correlates of Neocortical Ripple and High-Gamma Oscillations during Spindles of Natural Sleep

Robert G. Averkin, Viktor Szemenyei, Sándor Bordé, and Gábor Tamás

Supplemental Information

1. Inventory of Supplemental Information

Figure S1 illustrates recording quality and duration of our methodologically demanding recordings and overall data supplemental Figure 1.

Figure S2 is essential to further explain Figures 2-3 by presenting the stability of spindle ripple and spindle high gamma cell behavior over the course of spindle showing the lack of phase precession or phase lag as the spindle progresses, and illustrates illustrates FS cell heterogeneity, clustering, spike triggered firing latencies and the distribution of firing relative to peaks of activity.

Figure S3 shows that FS cell groups are not a product of spindle heterogeneity.

Figure S4 extends Figure 4 in showing similar phenomena in spindle cycles with spikes (as opposed to without spikes in Figure 4).

Figure S5 illustrates the variability of firing distribution of layer II and III pyramidal cells.

Table S1 presents data measured for FS cell subgroups in detail.

Video S1, related to Figure 1 is a step by step demonstration of our drug free in vivo recordings.

2. Supplemental Items

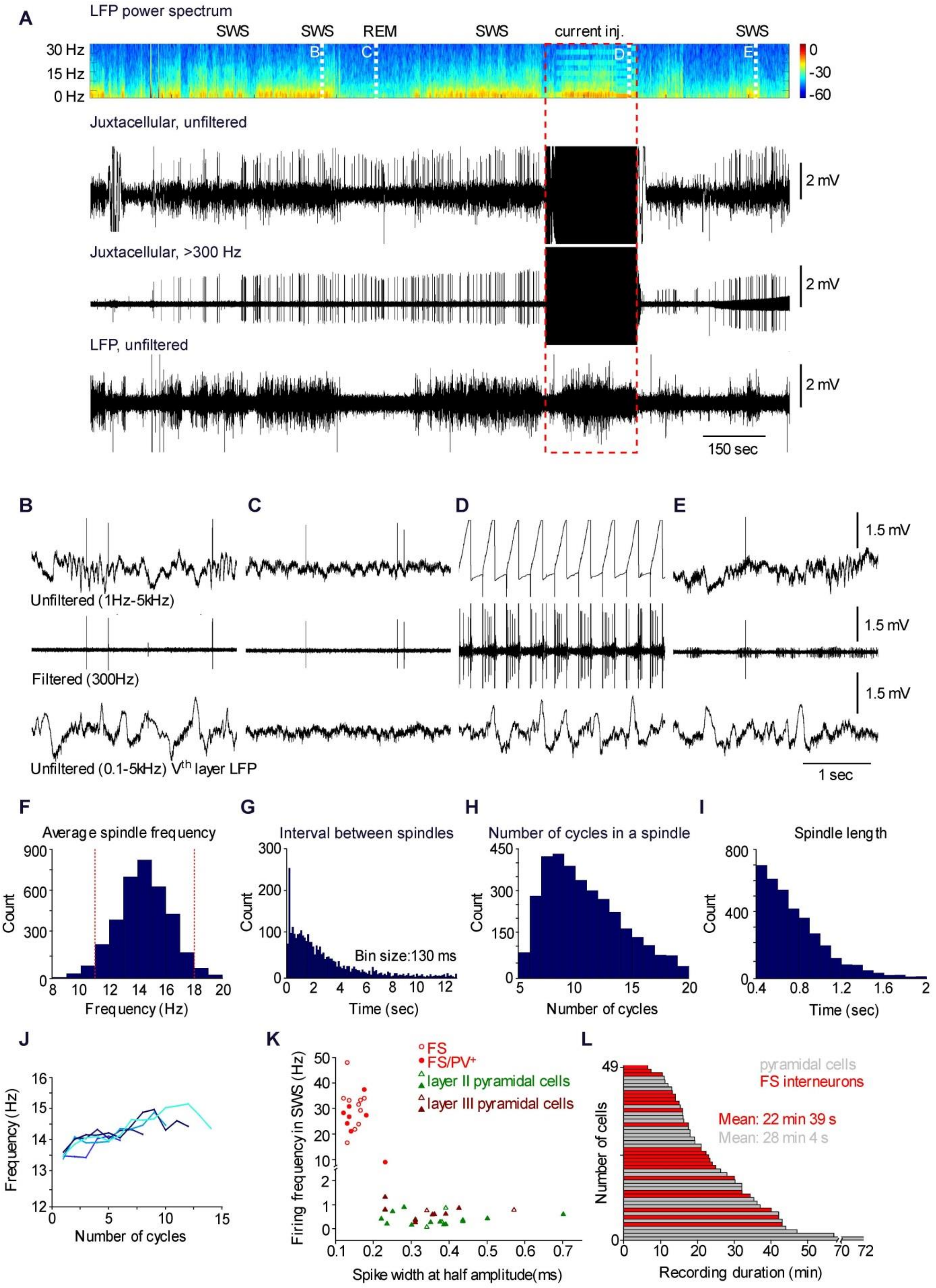

Figure S1, related to Figure 1. (**A**) Example of raw traces recorded in juxtacellular position to 2 layer II pyramidal cell with simultaneous local field potential (LFP) recording in layer V. Top panel, Power spectrum of the LFP. Top and middle trace, Wideband (top) and high pass filtered (middle) juxtacellular recording of the

layer II pyramidal cell activity. Bottom trace, Wideband raw trace of the layer V LFP. (**B-E**) Episodes of recordings presented on panel **A** shown at a detailed timescale during slow wave sleep (SWS, **B**, **E**), rapid eye movement sleep (REM, **C**) and positive current injection (current inj, **D**). (**F-J**) Distribution of internal spindle frequency (**F**), intervals separating successive spindles (**G**), the number of cycles per spindle (H) and of the length of spindles (**I**) in our overall dataset. (**J**) Instantaneous frequency of spindle cycles depending on the length of spindles. (**K**) Segregation of FS cells and pyramidal cells based on spike width and firing frequencies during SWS. (**L**) Length of continuous recordings at juxtacellular position to pyramidal cells and FS cells in freely behaving rats.

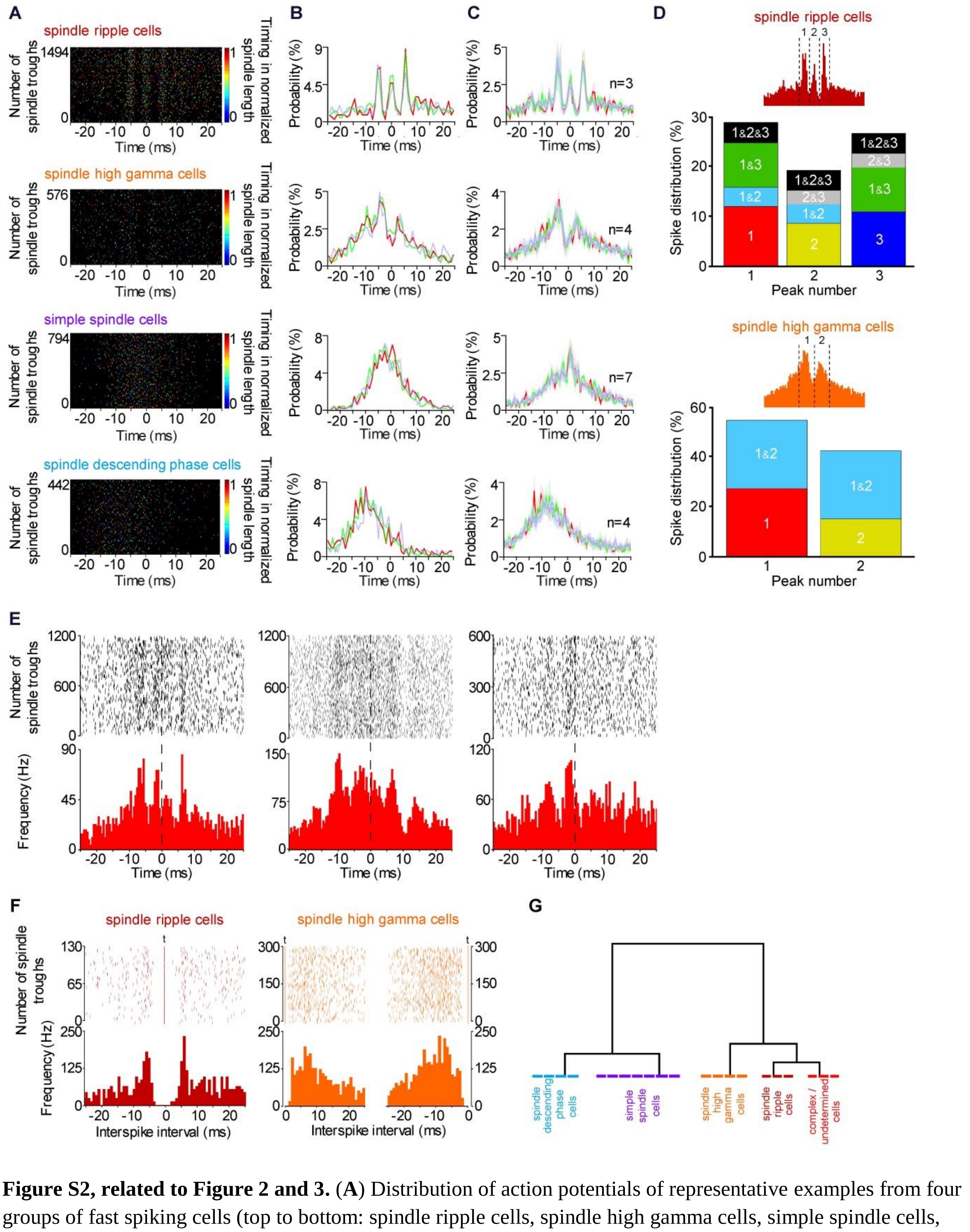

Figure S2, related to Figure 2 and 3. (**A**) Distribution of action potentials of representative examples from four groups of fast spiking cells (top to bottom: spindle ripple cells, spindle high gamma cells, simple spindle cells, spindle descending phase cells) color coded according to their timing relative to the normalized length of the actual ongoing spindle. The mixture of colors throughout the scattergram indicate the absence of phase precession or phase lag of spikes relative to spindle troughs. (**B**) High temporal resolution firing probability of the cells shown on **A** during the first (blue), second (green) and last third of spindle cycles (red). (**C**) Average high temporal resolution firing probability distributions of the four fast spiking cell populations during the first (blue), second (green) and last third of spindle cycles (red). Shadowed areas correspond to SEM. (**D**) Bottom, Examination of firing during single spindle troughs revealed that individual spindle high gamma cells fired

during the first (1) second (2) or both (1 & 2) peaks of overall activity (inset). Top, Single spindle ripple cells could be active during the first (1), second (2) or third (3) peak only, during two peaks (1 &2, 1 & 3, 2 & 3) and during all three peaks (1 & 2 & 3) of overall activity (inset). (**E**) Spindle trough related FS cells with complex high frequency timing of firing around spindle troughs (0 ms). These cells could not be classified as spindle ripple, spindle high gamma or simple spindle cells. (**F**) Spike triggered analysis of interspike intervals relative to action potentials of particular peaks of spindle-high gamma cells or spindle-ripple cells. Left, Interspike interval distribution of action potentials triggered (t) on spikes of the central peak of activity in a spindle ripple cell. Right, Latencies of spikes relative to action potentials in a spindle high gamma cell. The left and right panels show distributions triggered (t) relative to action potentials of the first and second peak of activity, respectively. (**G**) Hierarchical clustering using Ward's method based on the number of peaks in firing probability in ± 12.5 ms and ± 6.25 ms time windows centered at the trough of spindles resulted in five cell clusters corresponding to spindle ripple, spindle high gamma, simple spindle, spindle descending phase and complex / undetermined cells. Clustering was based on spindle phase relative to the trough.

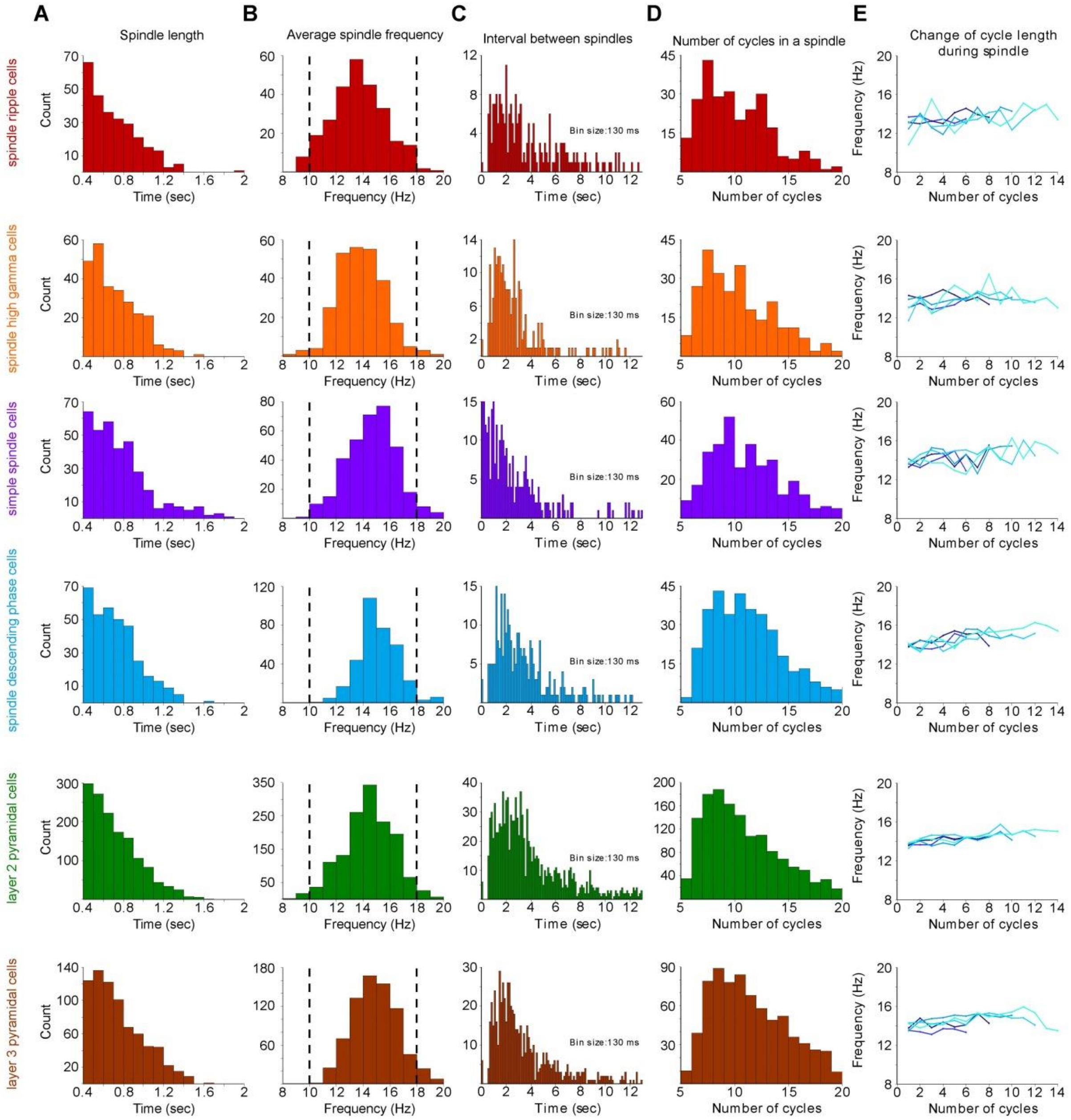

Figure S3, , related to Figure 2 and 3. Distribution of internal spindle length (**A**) average spindle frequency (**B**), intervals separating successive spindles (**C**), the number of cycles per spindle (**D**) in groups of FS cells and pyramidal cells. (**E**) Instantaneous frequency of spindle cycles depending on the length of spindles.

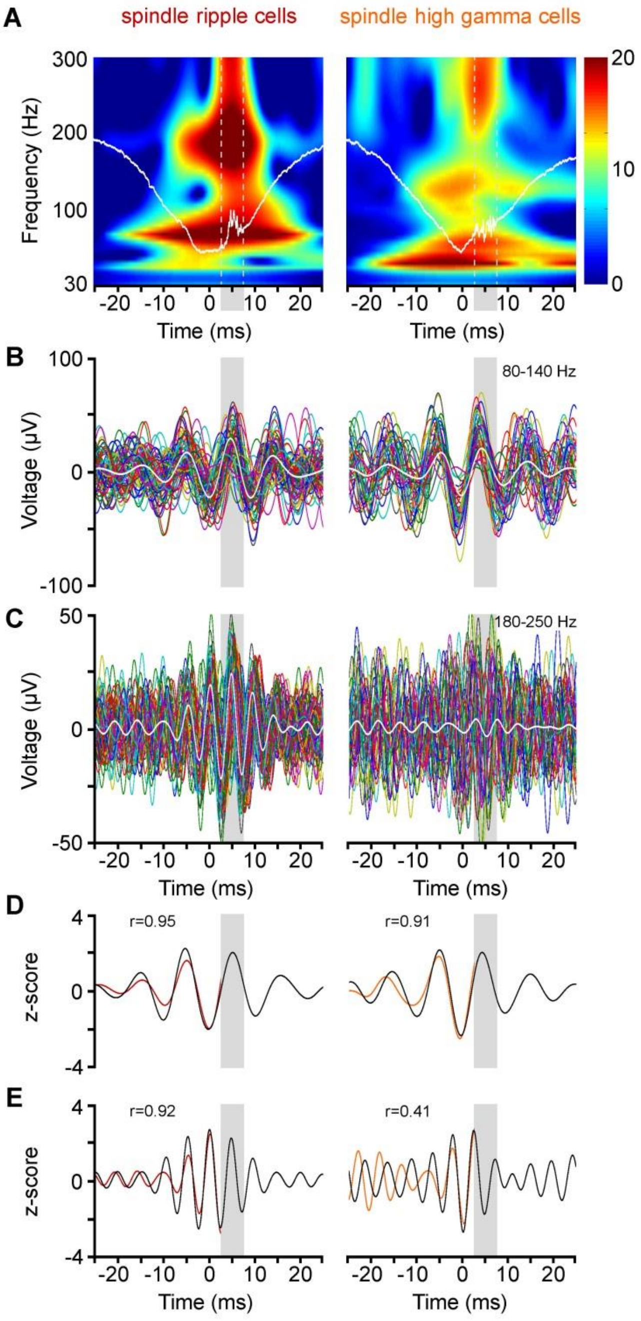

Figure S4, related to Figure 4. Presence of gamma and ripple band oscillatory field activity in spindle cycles with juxtacellularly detected firing. (**A**) Average wavelet spectra constructed from spindle troughs (timed at 0 ms) during which juxtacellular spikes were timed 2.5 to 7.5 ms (dashed lines and gray band) after the spindle trough in functional subgroups of FS cells. Both ripple-band and high gamma activity emerge around spindle troughs and prior to the spikes in recordings juxtacellular to spindle ripple cells, but activity predominantly in the high gamma range is characteristic to recordings juxtacellular to spindle high gamma cells. (**B-C**) Consecutive individual traces filtered 80-140 Hz (B) and 180-250 Hz (C) aligned according to spindle troughs (0 ms). Colored traces show all spindle troughs with spikes in the time window 2.5 to 7.5 ms after the trough (grey bands) available from a representative cell from each FS interneuron subgroup (same cell for B and C), averages of subgroups are shown in white. Oscillations in the high gamma range appear in both FS cell subgroups and the through of high gamma oscillations coincide with the spindle trough. Ripple oscillations are predominant in spindle ripple cells and the peak of ripple oscillations is at the trough of spindles. (**D, E**) FS cell subgroup dependent correlation between local field activity in spindle cycles with spikes (burgundy, orange; z-scored averages shown on panels B and C) and without spikes (black) bandpass filtered at 80-140 Hz and 180-250 Hz

recorded juxtacellularly to FS interneurons (r, Pearson correlation coefficients). Using the common time reference of spindle troughs, correlations were calculated for the time window of -25 to +2.5 ms relative to the trough between filtered and z-scored averages of local field potentials derived from spindle cycles with and without spikes.

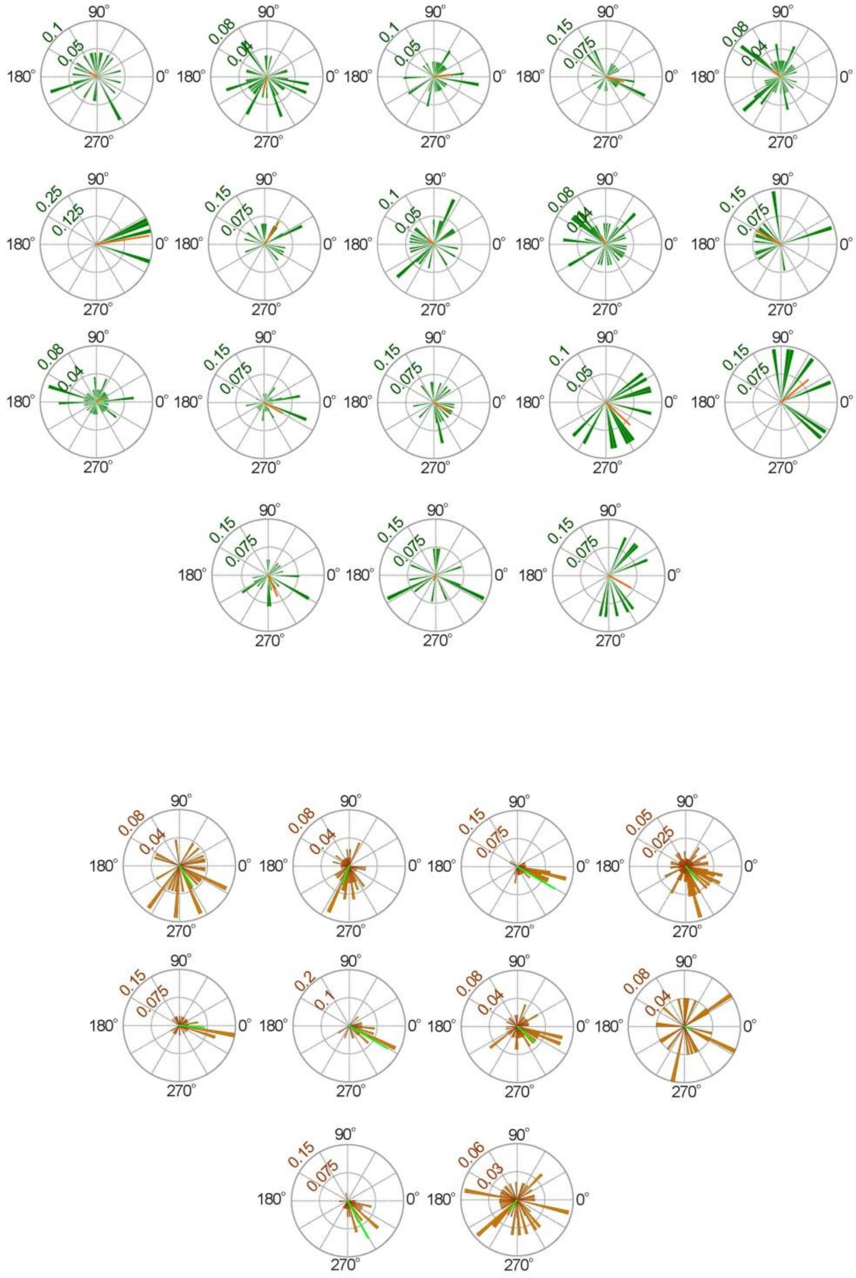

Figure S5, related to Figure 5. Circular plots of firing distribution of individual layer II (green, n=18) and layer III (brown, n=10) pyramidal cells.

Table S1, related to Figure 2 and 3. Electrophysiological characteristics of functional groups of FS cells recorded and labeled in drug-free conditions. Spike amp: spike amplitude; Spike width: spike half-amplitude width; DV: dorsal-ventral coordinates; ML: medial-lateral coordinates; AP: anterior-posterior coordinates; PV: parvalbumin.

Cell group and cell code	Spike amplitude[a] (mV)	Spike width[b] (ms)	Recording duration[c] (min:sec)	Firing rate (Hz) in SWS[d]	Number of spindle troughs		Mean vector direction[g] (degree)	Mean vector lengths[h]	Anatomical coordinates (mm)			PV	Number of peaks in spike timing (trough ± 6.25 ms)	Number of peaks in spike timing (trough ± 25 ms)
					all [e]	without spikes** [f]			DV	ML	AP			
Spindle ripple cells														
rat78d1t1c1	1.4	0.135	34:30	26.89	1138	241	9.31	0.284	160	0.83	3.24	+	3	3
rat95d3t2c1	1.0	0.165	42:00	29.45	1657	599	13.28	0.32	325	2.37	-4.08		3	3
rat121d1t3c1	2.2	0.18	13:16	27.5	1124	238	-17.83	0.335	250	2.5	-3.96		3	3
Mean	1.53	0.160	29:55	27.95	1306.3	359.3	1.06	0.31						
SD	0.61	0.023	14:54	1.34	303.8	207.6	14.05	0.03						
Median	1.40	0.165	34:30	27.50	1138.0	241.0	9.31	0.32						
Spindle high gamma cells														
rat119d1t1c2	1.1	0.12	22:30	34.21	701	95	6.35	0.3441	745	2.57	-3.76	+	2	2
rat72d1t1c1	0.25	*	14:00	32.7	646	102	-11.95	0.19	200	2.2	-4.0		2	2
rat131d2t1c2	0.85	0.14	15:00	21.2	580	67	-4.84	0.5	476	2.9	-3.72	+	2	2
rat35d1t1c4b	1.2	0.165	24:00	33.5	2151	246	-12.43	0.379	300	2.2	-2.72		2	2
Mean	0.85	0.142	18:52	30.40	1019.5	127.5	-5.12	0.35						
SD	0.43	0.073	05:06	6.17	756.0	80.4	7.25	0.13						
Median	0.98	0.140	18:45	33.10	673.5	98.5	-8.40	0.36						
Simple spindle cells														
rat36d1t3c2	1.5	0.15	07:40	21.95	263	28	9.31	0.554	450	2.0	4.0		1	1
rat133d1t1c1	1.34	0.245	43:00	9	1650	924	-3.22	0.64	364	2.0	-3.1	+	1	1
rat139d1t2c4	1.7	0.12	14:00	28.46	769	156	-16.67	0.26	594	2.47	-2.85	+	1	1
rat38d1t2c1	1	0.13	10:48	16.67	497	272	-7.2	0.33	350	3.0	-3.48		1	1
rat68d2t2c1	1.1	0.135	23:00	33.4	1684	225	24.92	0.34	200	2.4	-3.48		1	1
rat69d2t1c2	0.75	0.16	06:30	31.81	385	52	-12.19	0.424	230	2.65	-3.96		1	1
rat79d2t2c2	0.3	*	40:00	27	903	166	-9.45	0.725	400	2.0	4.0		1	1
Mean	1.10	0.157	20:43	24.04	878.7	369.3	-2.28	0.47						
SD	0.47	0.072	15:13	8.77	580.9	342.4	11.88	0.17						
Median	1.10	0.143	14:00	27.00	769.0	156.0	-7.20	0.42						
Spindle descending phase cells														
rat74d3t2c1	0.35	*	16:00	28.7	502	47	-47.8	0.6213	500	2.35	-3.27		0	1
rat157d1t1c3	0.6	0.13	23:26	48.34	2512	667	-42.37	0.265	580	2.1	-2.94		0	1
rat81d1t1c1	1.75	0.175	32:10	37.7	720	89	-22.57	0.32	306	2.4	-3.28	+	0	1
rat87d2t3c4	2.3	0.13	30:00	24.3	1502	175	-41.52	0.6	487	2.3	-3.4	+	0	1
Mean	1.25	0.145	25:24	34.76	1309.0	244.5	-40.49	0.45						
SD	0.93	0.076	07:17	10.63	909.7	286.7	8.70	0.19						
Median	1.18	0.130	26:43	33.20	1111.0	132.0	-41.95	0.46						

Complex (ungrouped) cells														
rat51d1t1c1	1.1	0.135	17:50	31	833	152	-2.5	0.13	222	2.3	-2.7	+	2	3
rat101d2t2c1	1.4	0.175	21:00	34.2	1384	164	-6.47	0.36	162	2.95	-3.6		2	3
rat126d1t2c2	1	0.16	25:00	24	1505	369	-4.93	0.31	293	2.4	-2.82		2	3
Mean	1.17	0.157	21:17	29.73	1240.7	228.3	-5.23	0.27						
SD	0.21	0.020	03:35	5.22	358.2	122.0	1.39	0.12						
Median	1.10	0.160	21:00	31.00	1384.0	164.0	-4.93	0.31						

* Spike half widths are not indicated due to low amplitude of the spikes.
** Time window without spikes was ± 25 ms around the troughs of spindle cycles.
The mean vector direction (MVD) of spindle descending phase cells significantly ([g] $p < 0.047$, random permutation test) preceded the MVD measured in either functional cell groups. No other parameters were significantly different between cell groups (Kruskal-Wallis test, [a] $p = 0.707$; [b] $p = 0.736$; [c] $p = 0.679$; [d] $p = 0.404$; [e] $p = 0.651$; [f] $p = 0.560$; [h] $p = 0.421$).
