# Supplementary figures and images for: Identified Cellular Correlates of Neocortical Ripple and High-Gamma Oscillations during Spindles of Natural Sleep
Source: Neuron. 2016 Nov 23;92(4):916–28. doi: 10.1016/j.neuron.2016.09.032 (PMC5130902; doi:10.1016/j.neuron.2016.09.032)

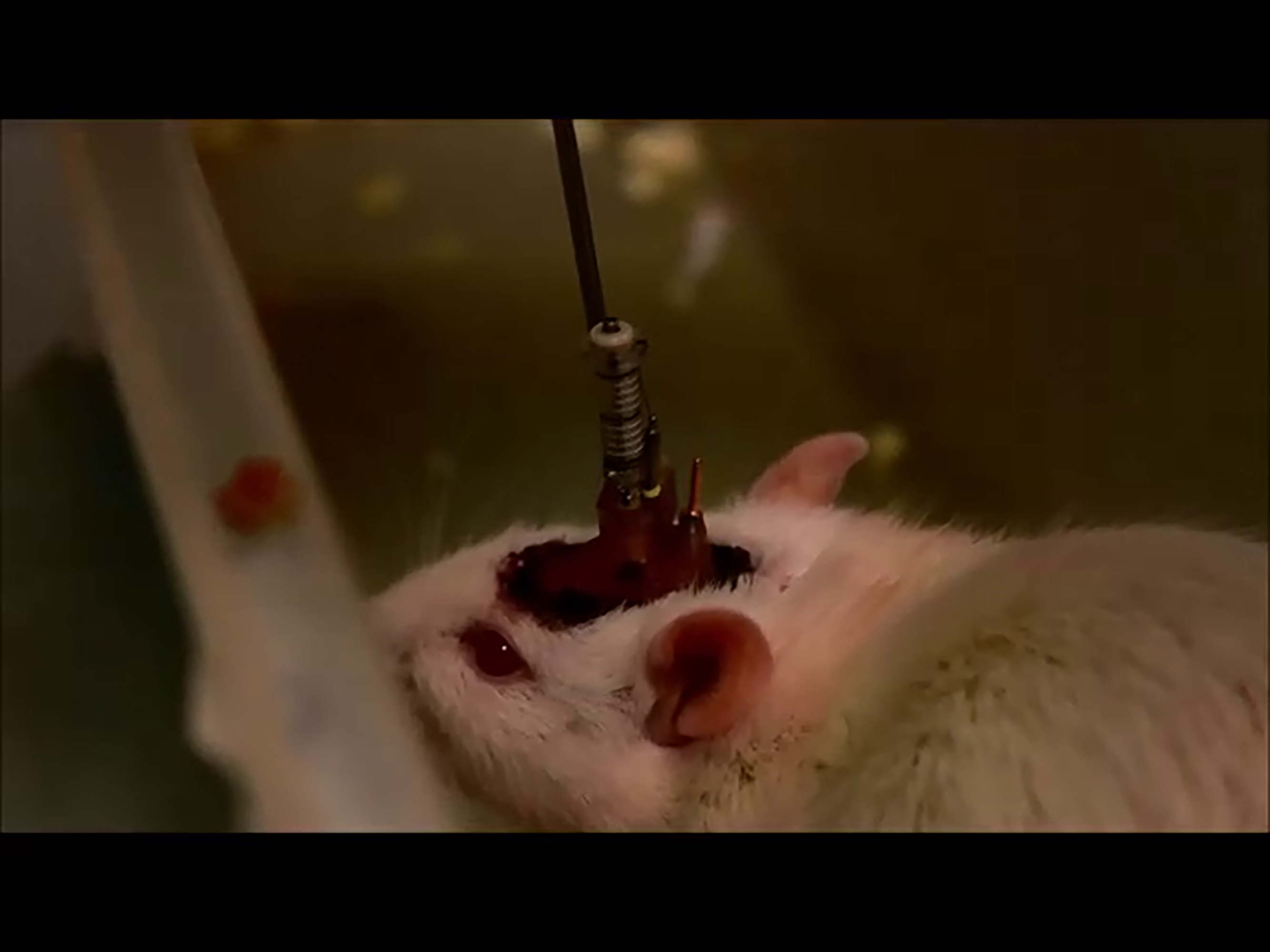

Supplement: Movie S1. Step-by-Step Demonstration of Our Drug-free In Vivo Recordings — Related to Figure 1. [file mmc2.jpg]
