# Supplementary material for: Identified Cellular Correlates of Neocortical Ripple and High-Gamma Oscillations during Spindles of Natural Sleep
Source: Neuron. 2016 Nov 23;92(4):916–28. doi: 10.1016/j.neuron.2016.09.032 (PMC5130902; doi:10.1016/j.neuron.2016.09.032)
Supplement: Document S2. Article plus Supplemental Information [file mmc3.pdf]

Article

Neuron

Identified Cellular Correlates of Neocortical Ripple and High-Gamma Oscillations during Spindles of Natural Sleep

Highlights

- Field potential events at ripple and high-gamma frequencies occur at spindle troughs
- Interneurons fire in phase with spindle ripple and spindle high-gamma oscillations
- Pyramidal cells fire sporadically and phase shifted relative to interneurons
- Spindle ripple events might provide neocortical reference states in mnemonic processes

Authors

Robert G. Averkin, Viktor Szemenyei, Sándor Bordé, Gábor Tamás

Correspondence

gtamas@bio.u-szeged.hu

In Brief

Averkin et al. find local oscillatory events in neocortical field potentials in time windows at troughs of spindle oscillations during sleep. Subgroups of fast-spiking interneurons fire at ripple and high-gamma frequencies in phase with the local field oscillations.

CrossMark

Averkin et al., 2016, Neuron *92*, 916–928
November 23, 2016 © 2016 The Authors. Published by Elsevier Inc.
http://dx.doi.org/10.1016/j.neuron.2016.09.032

CellPress

Identified Cellular Correlates of Neocortical Ripple and High-Gamma Oscillations during Spindles of Natural Sleep

Robert G. Averkin,[1] Viktor Szemenyei,[1] Sándor Bordé,[1] and Gábor Tamás[1,2,*]
[1]MTA-SZTE Research Group for Cortical Microcircuits, Department of Physiology, Anatomy and Neuroscience, University of Szeged, Közép fasor 52, Hungary
[2]Lead Contact
*Correspondence: gtamas@bio.u-szeged.hu
http://dx.doi.org/10.1016/j.neuron.2016.09.032

SUMMARY

Ultra-high-frequency network events in the hippocampus are instrumental in a dialogue with the neocortex during memory formation, but the existence of transient ~200 Hz network events in the neocortex is not clear. Our recordings from neocortical layer II/III of freely behaving rats revealed field potential events at ripple and high-gamma frequencies repeatedly occurring at troughs of spindle oscillations during sleep. Juxtacellular recordings identified subpopulations of fast-spiking, parvalbumin-containing basket cells with epochs of firing at ripple (~200 Hz) and high-gamma (~120 Hz) frequencies detected during spindles and centered with millisecond precision at the trough of spindle waves in phase with field potential events but phase shifted relative to pyramidal cell firing. The results suggest that basket cell subpopulations are involved in spindle-nested, high-frequency network events that hypothetically provide repeatedly occurring neocortical temporal reference states potentially involved in mnemonic processes.

INTRODUCTION

Precise temporal structure is necessary for neuronal information transfer, and neuronal oscillations provide a temporal framework for this process (Klausberger and Somogyi, 2008; Salinas and Sejnowski, 2001; Singer and Gray, 1995). Network oscillations in the cerebral cortex cover a wide range of frequencies and dynamic interactions between relatively slower rhythms, and higher frequency gamma oscillations (40–70 Hz) are implicated in forming higher-order representations through a multitude of potential mechanisms (Engel et al., 2001; Isaacson and Scanziani, 2011; Tiesinga and Sejnowski, 2009). Transient oscillations at even higher frequencies (~200 Hz), called sharp-wave ripples, are characteristic of the CA1 region of the hippocampus (Buzsáki et al., 1992) and appear coincident with the reactivation of ensemble memories (Wilson and McNaughton, 1994). Sharp-wave-ripple-encoded, initial mnemonic representations in the hippocampus are followed by a consolidation stage performed in the neocortex (Born et al., 2006; Buzsáki, 1996; McClelland et al., 1995), but the existence of transient ~200 Hz network events in the neocortex, potentially generated locally or by hippocampal inputs, is not clear. Spindle oscillations (8–16 Hz) of waxing and waning amplitude detected in slow-wave sleep (SWS) throughout the cortex are suggested to temporally organize the hypothesized memory transfer between hippocampus and neocortex (Contreras et al., 1997; Diekelmann and Born, 2010; Huguenard and McCormick, 2007; Rasch and Born, 2013). Classically associated with alert states (Steriade, 2000), gamma, high-gamma, and ultra-high band (200–800 Hz) oscillations of local neocortical origin were also observed in sleep (Hasenstaub et al., 2005; Kandel and Buzsáki, 1997); thus, we asked whether spindle oscillations provide temporal reference for cellular and network correlates of neocortical high-frequency activity.

RESULTS

High-Frequency Network Events Occur Repeatedly around Spindle Troughs

Previous studies reported nesting of hippocampal ripples in parahippocampal/neocortical spindle activity in humans (Clemens et al., 2011; Staresina et al., 2015) and the presence of high-frequency oscillations near the trough of high-voltage spike-and-wave spindles phase coupled to the firing of suspected interneurons in epileptic rat neocortex (Kandel and Buzsáki, 1997). Thus, we tested whether the high-frequency structure of local population activity of the neocortex might be embedded into spindles during SWS in freely behaving animals. Wavelet analysis of local field potentials recorded simultaneously with tungsten electrodes in layer II/III and with electrodes placed juxtacellularly to layer II/III pyramidal cells revealed the presence of events at high-gamma and ripple frequencies (Figures 1A–1H; n = 4 animals, n = 775 simultaneously recorded spindle troughs without action potentials detected on either electrode). The amplitude of these local field events was modulated within spindle cycles (modulation index × 10^{-3}, 3.341and 4.169 versus 1.743 and 1.462 for high-gamma and ripple band on tungsten versus juxtacellular electrodes, respectively) with maximal amplitudes around spindle troughs (mean vector direction [MVD], –25.40°

 © 2016 The Authors. Published by Elsevier Inc.
This is an open access article under the CC BY license (http://creativecommons.org/licenses/by/4.0/).

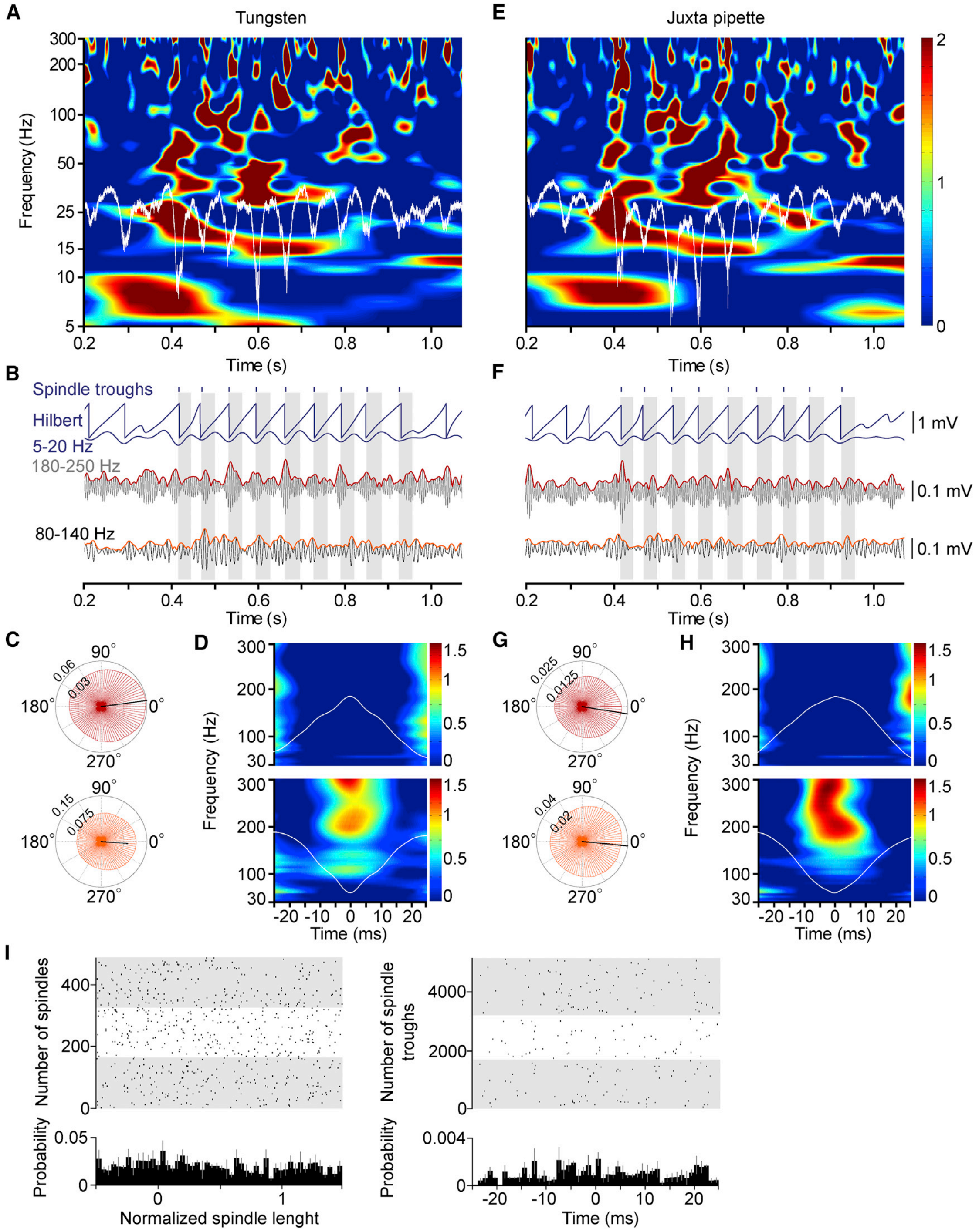

Figure 1. Spindle Nested High-Frequency Network Events Are Centered around Spindle Troughs during SWS

(A–H) Analysis of the same sleep spindle recorded simultaneously by a tungsten electrode in layer III (A–D) and a juxtacellular glass electrode placed adjacent to a layer III pyramidal cell (E–H).

(A and E) Wavelet analysis of the spindle (white trace, unfiltered) showing high-gamma and ripple frequencies emerging around troughs of the spindle.

(B and F) Hilbert transformation and phase-amplitude modulation at ripple (180–250 Hz, gray) and high-gamma (75–130 Hz, black) frequencies of the same spindle shown in (A) and (E). Gray bands indicate trough-to-peak halves of spindle cycles.

(C and G) Phase-amplitude coupling diagrams of high-gamma (orange) and ripple (red) frequency network events, and spindle waves (trough at 0°) indicate moderate coupling.

(legend continued on next page)

and –22.01°; mean vector length [MVL], 0.103 and 0.115 for high-gamma band and ripple band, respectively, on tungsten electrodes; MVD, –8.542° and –15.44°; MVL, 0.063 and 0.056 for high-gamma and ripple band, respectively, on juxtacellular electrodes). We then checked (in n = 3 animals) whether these spindle-nested high-gamma and ripple band events of the neocortex would be coupled to sharp-wave ripple activity simultaneously recorded in the dorsal CA1 region of the hippocampus. Sharp-wave ripples occurred throughout neocortical spindle oscillations and showed no apparent phase preference relative to spindle troughs (Figure 1I). There is no documented monosynaptic connection between any part of the hippocampus and the neocortical area we recorded from; thus, these results keep the possibility of neocortical generation of the high-frequency components of population events open. However, it is almost impossible to exclude the possibility of hippocampal influence on neocortical high-frequency network events based on single-site recordings from the hippocampus due to the complex functional topographical organization between the hippocampus and neocortex (Royer et al., 2010) and local generation and propagation of sharp-wave ripples along the septo-temporal axis of the hippocampus (Patel et al., 2013).

Heterogeneous Contribution of Fast-Spiking Cells to High-Frequency Events during Spindles

Cortical interneurons entrain postsynaptic neuron populations and contribute to oscillating cortical networks at various frequencies (Buzsáki and Draguhn, 2004; Klausberger and Somogyi, 2008; McBain and Fisahn, 2001). In search of underlying network mechanisms of high-frequency population events occurring repeatedly around spindle troughs described above, we set out to record the firing behavior of identified interneurons and principal cells in relation to spindle oscillations and high-frequency field events in freely behaving animals. We applied a drug-free installation of a pipette microdrive assembly (modified after Korshunov, 1995) for juxtacellular recording and labeling (Burgalossi et al., 2011; Pinault, 1996) of supragranular interneurons and pyramidal cells from dorsal cortex (parietal and secondary motor areas) during natural sleep in Wistar rats well adapted to experimental conditions (n = 35). Juxtacellular recording periods lasted for 26 min, 7 s ± 13 min, 30 s (Figure S1, available online) spanning several episodes of natural sleep. We involved sleep spindles (n = 3,370) in the analysis with spindle lengths of 0.4–2 s (0.764 ± 0.294 s) and with spindle oscillation frequencies between 10 and 18 Hz (14.46 ± 1.567 Hz); the probability for observing spindles with these properties was 0.317 ± 0.166 Hz during SWS epochs (Figures S1F–S1J). Fast-spiking (FS) interneurons (n = 21) had narrow action potentials (spike amplitude halfwidth, 0.153 ± 0.061 ms; Figures 2B, 3B, and S1K; Table S1) and a mean firing frequency of 28.67 ± 8.02 Hz during SWS, which dropped to 19.85 ± 11.56 Hz in REM sleep (n = 8; unpaired t test, $p < 0.027$) accompanied by inter-spike interval distributions that peaked at 5.20 ± 1.82 ms and 9.7 ± 2.97 ms, respectively ($p < 0.001$). Attempts to juxtacellularly label the recorded FS cells by application of positive current pulses (2–18 nA, 150–250 ms duty cycle) resulted in successful recovery of nine FS cells with axonal reconstruction possible from seven cells (Figures 2C, 2F, 2H, and 3C). All recovered interneurons showed morphological features of basket cells based on terminals surrounding postsynaptic somata, with immunocytochemistry detecting parvalbumin in eight labeled cells.

Rhythmic activity of all FS cells was observed during spindle episodes of SWS locked to a particular phase of spindle cycles (Figures 2D, 2E, 2G, 3B, and 3D). According to the firing phase preference in spindle cycles, the distribution of mean vectors was non-uniform (Hodges-Ajne test, $p < 0.001$) and non-unimodal (Kuiper's two-sample test, average p = 0.771). Thus, rhythmic cells were tentatively grouped as spindle-trough-related FS interneurons (n = 17; MVL, 0.326; MVD, 355° ± 18°; Rayleigh z value, 1,355; Figure 2) and spindle descending-phase-related cells (n = 4; MVL, 0.402; MVD, 315° ± 7°; Rayleigh z value, 3,183; Figure 3). Thus, supporting earlier results from anesthetized (Hartwich et al., 2009) and drug-free sleep (Gardner et al., 2013; Peyrache et al., 2011), we found that FS interneurons are heterogeneously recruited relative to spindle cycles of natural sleep, suggesting variable intracortical versus thalamocortical excitatory input dominance (Beierlein et al., 2003; Cruikshank et al., 2007; Gardner et al., 2013; Isaacson and Scanziani, 2011; Peyrache et al., 2011; Puig et al., 2008) arriving to spindle-trough-related and spindle descending-phase interneurons, respectively.

The trough of the spindle cycle represents a strongly depolarized state of the cortical network (Buzsáki et al., 2012; Einevoll et al., 2013). For a better understanding of the contribution of FS interneurons to such depolarized network states, we further analyzed the temporal structure of their suprathreshold activity at a higher temporal resolution of millisecond precision, which required continuous recordings containing >250 spindle troughs. Out of 17 spindle-trough-related cells, n = 7 cells exhibited a single peak of activity on peri-event firing histograms centered near the spindle trough (simple spindle trough cells; MVL, 0.416; MVD, 357.43°; Rayleigh z value, 2,051; Rayleigh test $p < 0.0001$; Figure 2G). In addition, the activity of n = 4 FS interneurons showed two peaks, –4.71 ± 0.65 ms prior to and 3.52 ± 0.66 ms after the spindle trough, separated by a relative drop in firing frequency, resulting in periodic activity of 122 ± 3 Hz corresponding to high-gamma frequencies (spindle high-gamma cells; Figure 2E). Furthermore, n = 3 cells recorded in three animals showed a stereotypic triple-peaked activation pattern at frequencies of 205 ± 23 Hz, similar to the frequency

(D and H) Wavelet spectra triggered relative to spindle peaks (timed at 0 ms, top panels) and troughs (timed at 0 ms, bottom panels) show spindle-trough-centered emergence of ripple band (spindle ripple) and high-gamma (spindle high gamma) oscillations.

(I) Sharp-wave ripples in the dorsal CA1 region of the hippocampus are not preferentially timed relative to simultaneously recorded neocortical spindle oscillations. Left: timing of the peak of maximal amplitude cycles in hippocampal ripples (dots) during consecutive spindles of normalized length from three animals (gray and white bands) with the average and SD of ripple probability shown on the histogram. Right: timing of hippocampal ripples (dots) during consecutive spindle troughs from three animals (gray and white bands) with the average and SD of ripple probability shown on the histogram relative to the spindle trough timed at 0 ms (bin width, 0.78 ms).

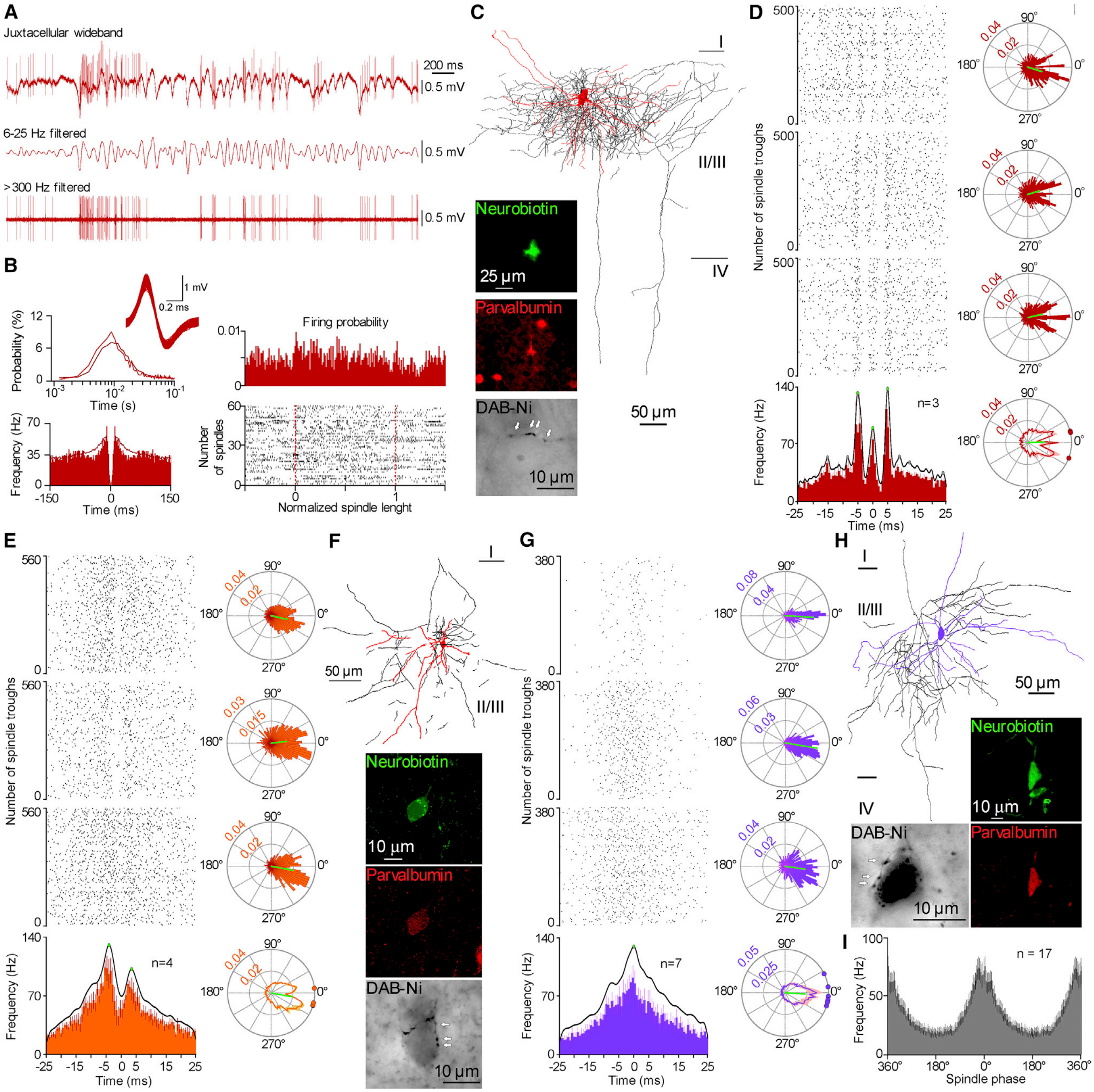

Figure 2. High-Frequency Contribution of Identified FS Interneurons to the Trough of Spindle Oscillations during Natural Sleep

(A) Juxtacellularly recorded activity of an identified spindle ripple cell (top, wideband; middle, filtered for spindles; bottom, filtered for spikes).

(B) Normalized interspike interval distribution (top left, burgundy for overall, red for spindles; inset, time course of single spikes) and autocorrelogram (bottom left, burgundy for overall, red for spindles), and firing distribution of the cell during spindles of normalized length (right).

(C) Top: reconstruction of the dendritic (red) and axonal (black) arborization of the spindle ripple cell. Insets: the neurobiotin-labeled cell expressed parvalbumin and showed features of a basket cell with DAB-Ni containing axonal terminals (arrows) decorating an unlabeled soma.

(D) Top left: peri-trough raster plots of firing of individual spindle ripple cells (top three panels; the top panel shows the cell presented in A–C) and average firing frequency distribution (bottom left; bin width, 0.78 ms) with three peaks of activity centered around the spindle trough shown at 0 ms determined by kernel smoothing and peak detection. Data are based on successive spindle troughs recorded in SWS. Right: cumulative circular plots of firing probability in single cycles of spindles for the three cells (trough at 0°) and their average circular plot of firing distribution (bottom) with vectors of individual cells at the perimeter. Error bars represent SEM.

(E–H) Spindle high-gamma cells (E and F) and simple spindle trough cells (G and H) are presented along the same logic as spindle ripple cells in (D) and (C). Error bars in (E) and (G) represent SEM.

(I) Average firing probability of all spindle-trough-related FS cells tuned to the trough (0°) of spindle cycles, including spindle ripple, spindle high-gamma, and simple spindle trough cells. Error bars represent SEM.

of hippocampal ripples. The central peak of the triple-peaked, ripple-like pattern was close (–0.09 ± 0.34 ms) to the trough of spindle waves, which was preceded and followed by two additional peaks of activity before (–4.86 ± 0.81 ms) and after (4.98 ± 0.22 ms) the spindle trough (spindle ripple cells; Figure 2D). The remaining n = 3 spindle-trough-related FS cells showed complex timing of firing with uneven intervals between peaks (Figure S2E). Hierarchical clustering using Ward's method based on the number of peaks in firing probability relative to absolute spindle phase in ± 25 ms and ± 6.25 ms time windows centered at the trough of spindles resulted in five cell clusters corresponding to spindle ripple, spindle high-gamma, simple spindle, spindle descending-phase, and complex cells, as detailed above (Figure S2G).

Although the frequencies of repeated peaks of firing in spindle high-gamma and spindle ripple cells were different ($p < 0.034$, Kruskal-Wallis test), the first peaks of spindle high-gamma and spindle ripple cells appeared synchronously before the spindle trough, suggesting a common excitatory input followed by anti-phase firing probabilities in the two cell groups at the spindle trough and close to in-phase activity later on. Spike-triggered analysis of interspike intervals relative to action potentials of particular peaks of spindle high-gamma cells or spindle ripple cells corroborated that firing of FS cells during spindle oscillations is enriched with subpopulation-specific high-frequency components centered at spindle troughs (Figure S2F). Examination of firing during single spindle troughs revealed that individual spindle high-gamma cells fired during the first (27.21% ± 5.6%), second (14.96% ± 5.9%), or both (27.40% ± 11.2%) peaks of overall activity (Figure S2D). Single spindle ripple cells could be active during the first (11.9% ± 2.1%), second (8.51% ± 4.5%), or third (10.82% ± 4.5%) peak only; during two peaks (3.84% ± 0.87%, 8.89% ± 1.2%, 2.71% ± 1.3% for first + second, first + third, and second + third, respectively); and during all three peaks of activity (4.08% ± 3.2%; Figure S2D). The two (spindle high-gamma cells) or three (spindle ripple cells) peaked distributions of action potentials around troughs remained stable throughout successive waves of spindles; thus, phase precession or phase lag of spikes was not observed (Figures S2A–S2C). Our results identify high-gamma- and ripple-frequency-tuned episodes of FS cell firing.

Suprathreshold activity of FS cells at ripple and high-gamma frequencies is nested in spindle oscillations and occurs repeatedly at successive spindle troughs. Interestingly, subpopulations of parvalbumin-immunoreactive interneurons are heterogeneously associated with high-frequency (epsilon) oscillations in the hippocampus (Varga et al., 2014), and parvalbumin-containing basket cells, similar to spindle high-gamma and spindle ripple cells, are linked by gap junctions that support the synchronization at high frequencies found here (Atallah and Scanziani, 2009; Cardin et al., 2009; Galarreta and Hestrin, 1999; Gibson et al., 1999; Meyer et al., 2002; Sohal et al., 2009). Spindle events are known to vary by sleep stage and brain area (Terrier and Gottesmann, 1978); therefore, we analyzed the properties of spindles recorded for each functional sub-class of FS cells (Figure S3). None of the spindle parameters measured were significantly different between the functional sub-classes of neurons; thus, the observed differences in spindle-associated physiological patterns were not attributable to differences in the properties of spindles recorded for each cell group.

Juxtacellular electrodes are sensitive to signals of a relatively wide frequency range covering spikes of individual neurons, as well as local field potentials generally understood as a representation of neural populations (Buzsáki et al., 2012). All of our juxtacellular recordings from interneurons contained sufficient numbers of spindle troughs without spikes (Table S1), allowing the search for the presence of ongoing high-frequency activity in the juxtacellular field potential around the cell being recorded. Both ripple band and high-gamma field activity emerged around spindle troughs in recordings juxtacellular to spindle ripple cells (Figure 4A), but activity predominantly in the high-gamma range was characteristic of recordings juxtacellular to spindle high-gamma, simple spindle, and spindle descending-phase cells (Figure 4A). The trough of high-gamma oscillations coincided with the spindle trough in all FS cell subgroups, ripple oscillations were predominant in spindle ripple cells, and the peak of ripple oscillations was at the trough of spindles (Figures 4B and 4C). We tested correlations between the firing of cell groups and the juxtacellular field activity using bandpass-filtered kernel-smoothed firing distributions z scored together with field oscillations in time windows (± 25 ms) around the spindle trough (Figures 4D and 4E). In the ripple-frequency band, significant correlation ($r = 0.83$, Pearson correlation; $p < 0.001$, random permutation test) between juxtacellular firing and field was restricted to spindle ripple cells with in-phase timing. In addition, the firing of spindle ripple cells ($r = 0.88$, $p < 0.001$) and spindle high-gamma cells ($r = 0.93$, $p < 0.001$) showed in-phase correlation to the juxtacellular field, and the firing of simple spindle cells was negatively correlated ($r = -0.86$, $p < 0.002$) due to an anti-phase relationship relative to the field. Furthermore, we also tested the presence of gamma and ripple band oscillatory field activity in spindle cycles in which the cells fired (Figure S4). Spindle cycles with spikes were involved in the analysis in case the spike was timed at the last peak of modulated activity in spindle ripple or spindle high-gamma cells, i.e., during the third peak in spindle ripple cells and during the second peak in spindle high-gamma cells (2.5–7.5 ms after the spindle trough in both cell groups). This allowed a time window for analyzing juxtacellular field oscillations without the influence of the subsequent spike showing that ripple band and high-gamma band oscillations were present prior to the spike in spindle ripple cells and that high-gamma band oscillations are characteristic of spindle high-gamma cells prior to the spike in spindle troughs (Figures S4A–S4C). The power of these spike-preceding oscillations was not significantly different from those measured in the same relative time windows in spindle troughs without spikes (Wilcoxon paired signed-rank test, spindle ripple cells, $p = 0.75$ and $p = 0.25$; spindle high-gamma cells, $p = 0.625$ and $p = 0.125$ for 80–140 and 180–250 Hz, respectively). In addition, oscillations in spindle troughs with and without spikes tested between –25 and 2.5 ms relative to the trough were correlated in phase at ripple ($r = 0.92$, Pearson correlation; $p < 0.001$, random permutation test) and high-gamma ($r = 0.95$, $p < 0.001$) bands in spindle ripple cells and at high-gamma frequencies in spindle high-gamma cells ($r = 0.91$, $p < 0.001$; Figures S4D and S4E). Thus, suprathreshold activity of functional subgroups of FS cells

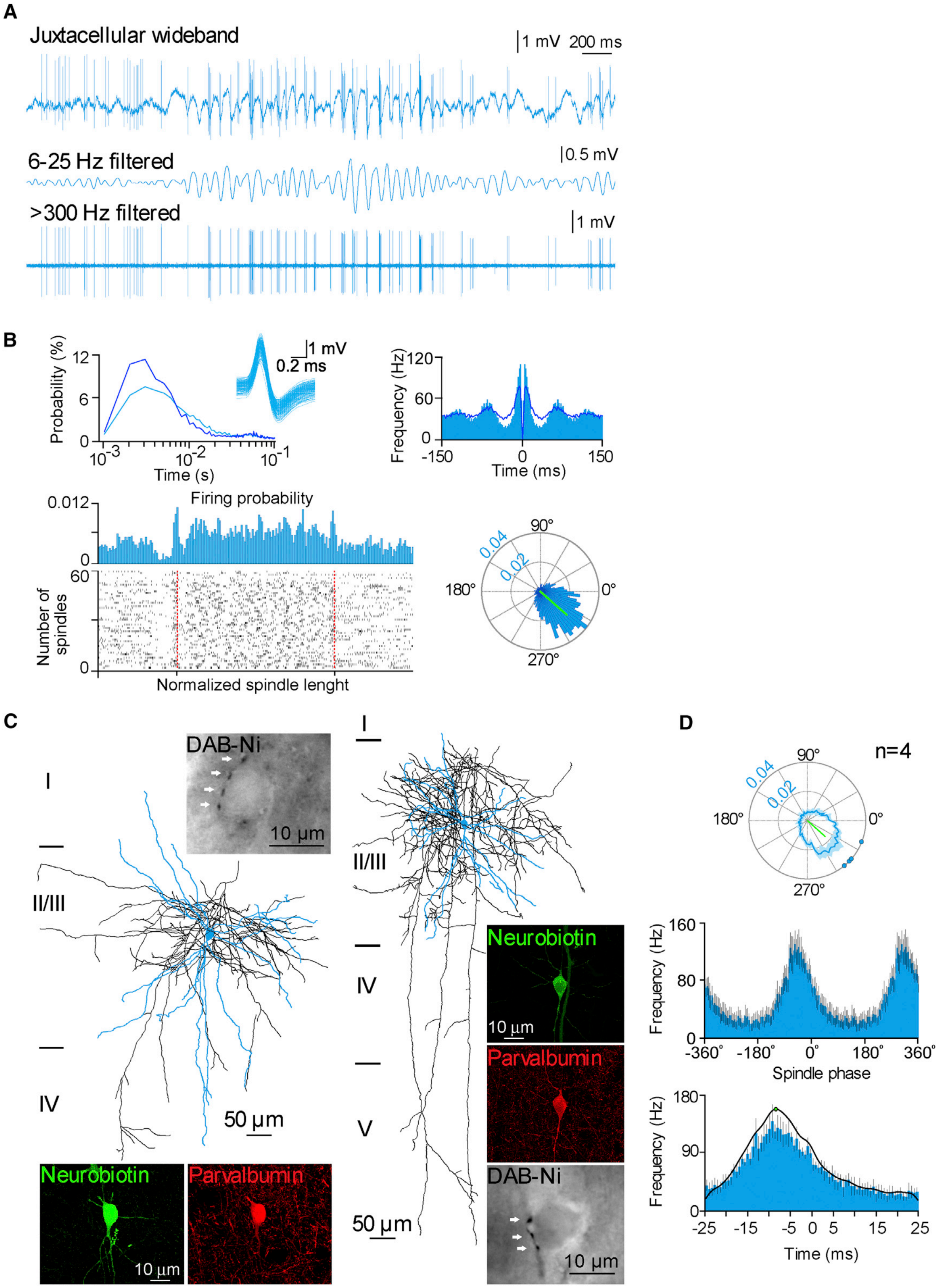

Figure 3. Contribution of FS Interneurons to the Descending Phase of Spindle Cycles

(A) Juxtacellularly recorded activity of an identified spindle descending-phase cell (top, wideband; middle, filtered for spindles; bottom, filtered for spikes).

(B) Interspike interval distribution (top left, dark blue for overall, light blue for spindles; inset. time course of single spikes), autocorrelogram (top right), and firing distribution of the cell during spindles of normalized length (bottom right). Bottom right: circular plot of firing probability of the cell in single cycles of spindles (trough at 0°).

(legend continued on next page)

is differentially embedded into high-frequency oscillations of local field potentials, and firing and local field potentials show parallel spectral and phase characteristics juxtacellular to spindle ripple and spindle high-gamma cells.

Different Contribution of Superficial and Deep Supragranular Pyramidal Cells to Spindle-Trough-Related Activity

A dynamic interplay between local pyramidal cells and interneurons—especially parvalbumin-containing basket cells—has been suggested as a potential mechanism resulting in gamma and higher-frequency oscillations (Cardin et al., 2009; Csicsvari et al., 1999; English et al., 2014; Fisahn et al., 1998; Hasenstaub et al., 2005; Sohal et al., 2009; Stark et al., 2014). Thus, we asked how the firing of pyramidal cells is recruited in relation to the fine-scale temporal orchestration of the FS interneurons during spindles. We searched for potential correlations between firing behavior and laminar placement of pyramidal cells following canonical models (Douglas and Martin, 2004) and reports of altered firing in superficial versus deep pyramidal cells within a cortical layer (Valero et al., 2015). The laminar position of pyramidal cells (n = 28) was determined based on anatomical recovery (n = 22; Figure 5A) or according to electrode depth measurements (n = 4, layer II; n = 2, layer III cells). Layer III pyramidal cells (n = 10) fired at higher overall frequencies (0.685 ± 0.296 Hz) compared to sporadically firing layer II cells (n = 18, 0.412 ± 0.272 Hz; unpaired t test, $p = 0.031$). Moreover, layer III pyramidal cells preferred firing before the troughs of spindle cycles (n = 10, 1.62 ± 0.74 Hz and 0.61 ± 0.35 Hz before and after; paired-samples t test, $p < 0.001$; Figures 5B–5D), while layer II pyramidal cells showed no difference before and after spindle troughs (n = 18, 0.69 ± 0.47 Hz and 0.63 ± 0.48 Hz before and after; $p = 0.442$, paired-samples t test). Relative to FS cells, however, both pyramidal populations showed an order of magnitude smaller average firing frequency during spindles ($p < 0.001$, ANOVA), firing only in 2%–15% of the spindle cycles that demanded the collection of high numbers of spindle troughs from layer III and II pyramids (n = 9,900 and n = 18,200, respectively) for the construction of spindle phase firing histograms. Layer III pyramidal cells showed firing preference to the descending phase of spindle cycle (MVL, 0.4056; MVD, –44.81°; Rayleigh z value, 57.2269; Rayleigh test, $p < 0.0001$; Figures 5B and 5C), while phase preference was weaker in layer II pyramidal cells (MVL, 0.1253; MVD, –20.72; Rayleigh z value, 3.0949; Rayleigh test, $p = 0.045$; independent-samples t test; $p = 0.0085$). Further differences emerged between the two pyramidal cell populations in the higher-frequency domains around the spindle trough. Layer III cells showed a first smaller peak at –6.8 ms and a second bigger peak at –2.8 ms; then firing remained suppressed through the immediate spindle trough, with firing reemerging with a third peak at 2.6 ms. On the other hand, the population of layer II pyramidal cells showed two small peaks following each other at –3.9 and 5.3 ms in the high-gamma frequency range with a silent period around the spindle trough. Overall, cell-to-cell variability of vectors and rates of firing were hallmarks of layer II cells in contrast to more coherent activity in layer III cells (Figures 5C and S5), in agreement with models of canonical microcircuits suggesting different roles for deep versus superficial pyramidal cells in neocortical processing (Douglas and Martin, 2004).

Finally, we searched for correlations between the firing of layer II and III pyramidal cells and the field activity of the surrounding microcircuit and compared the results to those obtained juxtacellular to FS cells. Both ripple band and high-gamma field activity emerged around spindle troughs in recordings juxtacellular to layer III pyramidal cells, and activity predominantly in the high-gamma range was characteristic of recordings juxtacellular to layer II cells (Figure 6A). The trough of high-gamma oscillations was timed at the spindle trough in both layer II and III pyramidal cells. Ripple oscillations characterized the population of layer III pyramidal cells, and the trough of ripple oscillations was coincident with the trough of spindles (Figures 6B and 6C). Correlations were also revealed between z-scored, bandpass-filtered, kernel-smoothed firing of pyramidal cells and z-scored field activity. Distribution of firing and field oscillations (Figures 6D and 6E) were correlated around (± 25ms) spindle troughs juxtacellular to both layer II ($r = 0.76$, Pearson correlation; $p < 0.01$, random permutation test) and layer III ($r = 0.78$, $p < 0.03$) pyramidal cells in the high-gamma frequency range with in-phase timing. Significant correlation ($r = 0.83$, $p < 0.001$) between juxtacellular firing and field in the ripple frequency band was observed in layer III cells, with a phase shift of –0.96 ms. Accordingly, the firing of pyramidal cells is heterogeneously related to the surrounding field activity.

Correlations between spindle-nested high-gamma and ripple band field events and the suprathreshold activity of pyramidal cells and interneurons, together with the timing of peaks in the firing of different groups of pyramidal cells and FS interneurons, suggest a potential interplay of FS and pyramidal cell subpopulations around spindle troughs. In particular, sequential activation of layer III pyramidal cells followed by spindle ripple cells with latencies of ~1–3 ms is shown above, suggesting contribution to ~200 Hz network episodes. Indeed, reciprocal monosynaptic coupling is characteristic of basket cells and layer III pyramids (Isaacson and Scanziani, 2011; McBain and Fisahn, 2001), and alternating action of pyramidal and basket cells has been suggested during hippocampal ripples (English et al., 2014; Stark et al., 2015). Compared to ripple band network events likely to involve spindle ripple interneurons and layer III pyramidal cells, spindle high-gamma oscillations with correlated firing were observed in a wider range of pyramidal cells and interneurons, suggesting synchronized firing of these neuron populations during network activity in the high-gamma range.

(C) Left: reconstructions of the dendritic (blue) and axonal (black) arborization of spindle descending-phase cells. The cell on the left is shown in (A) and (B). Insets: the neurobiotin-labeled cells expressed parvalbumin and showed features of basket cells with DAB-Ni containing axonal terminals (arrows) decorating unlabeled somata.

(D) Average circular plot of firing frequency with vectors of individual cells at the perimeter (top) and firing probability distribution (middle) in single spindle cycles. Bottom: the average firing probability of all spindle descending-phase-related FS cells tuned to the descending phase of the spindle cycle with a single peak of activity determined by kernel smoothing and peak detection. Error bars represent SEM.

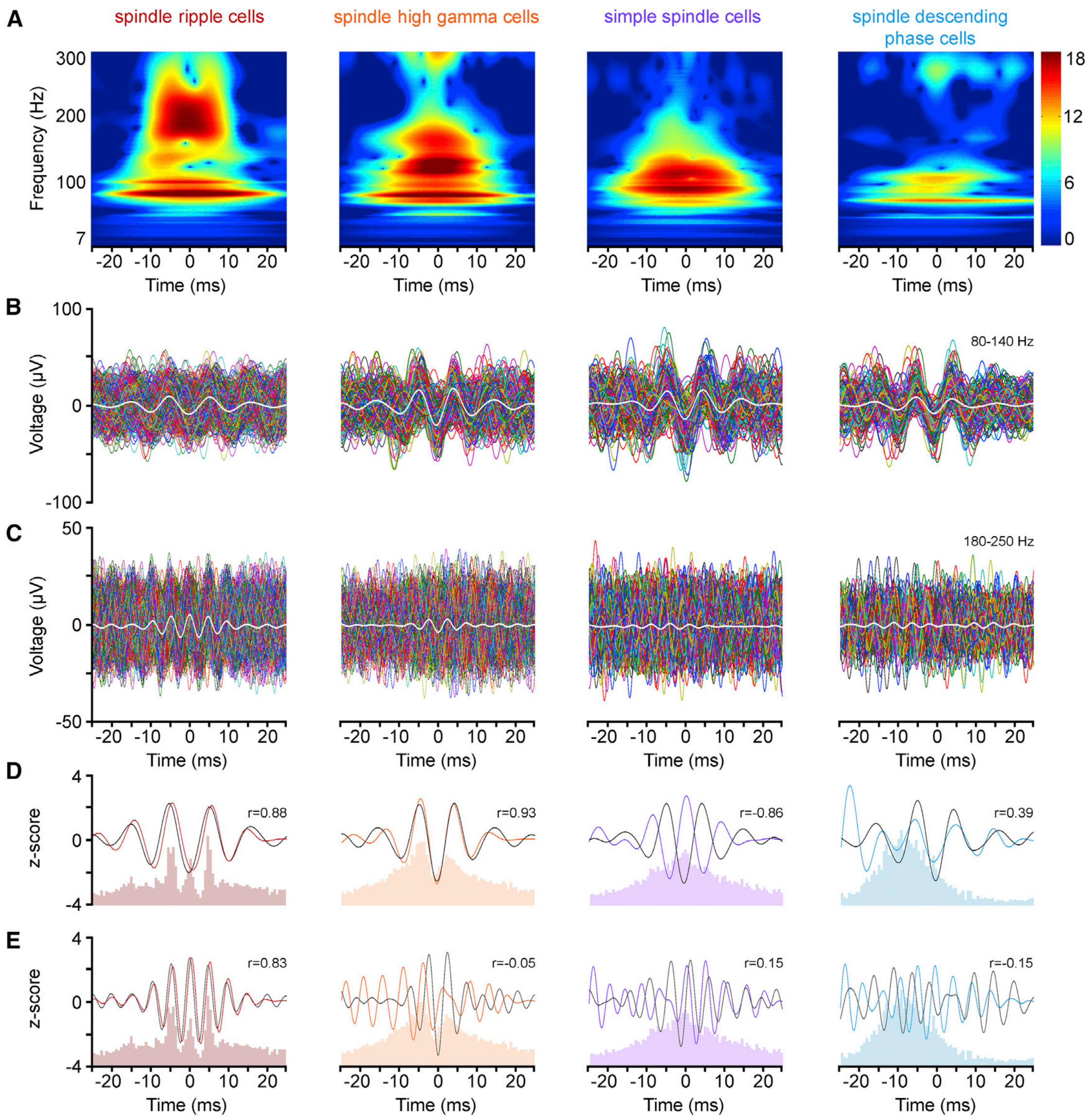

Figure 4. High-Frequency Local Field Potentials at the Trough of Spindle Oscillations Recorded Juxtacellularly around Identified FS Interneurons Are Variable

(A) Average wavelet spectra constructed from spindle troughs (timed at 0 ms) during which juxtacellular spikes were absent in functional subgroups of FS cells. Both ripple band and high-gamma activity emerge around spindle troughs in recordings juxtacellular to spindle ripple cells, but activity predominantly in the high-gamma range is characteristic of recordings juxtacellular to other functional subgroups of FS cells (spindle high-gamma, simple spindle, and spindle descending-phase cells).

(B and C) Consecutive individual traces filtered 80–140 Hz (B) and 180–250 Hz (C) aligned according to spindle troughs (0 ms). Colored traces show all spindle troughs without spikes available from a representative cell from each FS interneuron subgroup (same cell for B and C); averages of subgroups are shown in white. Oscillations in the high-gamma range appear in all FS subgroups, and the trough of high-gamma oscillations coincides with the spindle trough. Ripple oscillations are predominant in spindle ripple cells and the peak of ripple oscillations is at the trough of spindles.

(D and E) Foreground: FS cell subgroup-dependent correlation between the firing (burgundy, orange, red, and blue; kernel-smoothed firing frequency distributions bandpass filtered at 80–140 Hz, D, and 180–250 Hz, E) and local field activity (black; z-scored averages shown in B and C) recorded juxtacellularly to FS interneurons (r, Pearson correlation coefficients). Background: average firing frequency distribution histograms of FS cell subgroups. Using the common time reference of spindle troughs, spikes were correlated with the oscillations shown in (A) and (B) recorded during spindle cycles without firing.

DISCUSSION

We found that subgroups of pyramidal cells and FS interneurons exhibit a complex firing pattern organized around a reset-like inhibition timed at the spindle trough. Based on correlative evidence, this reset is enforced on layer II and III pyramidal cells and spindle high-gamma interneurons as in-phase suppression of firing by the population of spindle ripple cells operating at

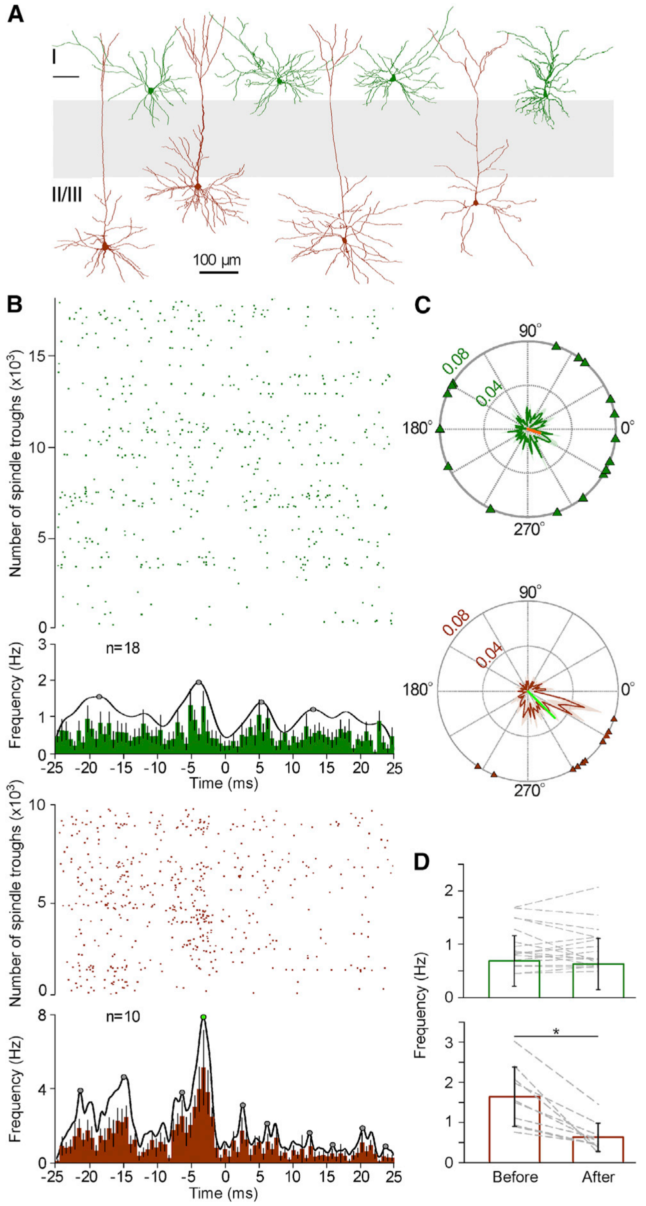

Figure 5. Differential Recruitment of Superficial Layer II and Deeper Layer III Pyramidal Cells during Spindle Troughs of Natural Sleep

(A) Dendritic reconstructions of juxtacellularly recorded superficial layer II (green) and deep layer III (brown) pyramidal cells. Cells with somata at cortical depth shown in gray were excluded from the study.

(B) Raster plots of firing (left) around the spindle trough (0 ms) with corresponding cumulative circular plots of firing distribution within single cycles of spindles (right) of individual layer II (n = 18) and layer III (n = 10) pyramidal cells and averages of firing frequency distributions around the spindle trough (bottom; bin width, 0.78 ms; error bars, SEM). Error bars represent SEM.

(C) Average circular plots of firing distribution (right) with vectors of individual cells at the perimeter indicate that deep layer III pyramidal cells predominantly fire just before the trough, in contrast to temporally scattered vectors of superficial layer II cells.

(D) Firing frequencies before and after spindle troughs were similar in layer II pyramidal cells, but dropped following the trough in deep layer III pyramidal cells (left). Error bars represent SEM.

200 Hz for three successive cycles centered at spindle troughs and possibly by simple spindle cells with a single, temporally wider peak of firing at spindle troughs. In addition, time windows prior to and after spindle troughs also exhibit population-dependent, highly structured firing in the network orchestrated to form spindle high-gamma and spindle ripple events in the local network. Sequential activity of layer III pyramidal cells and spindle ripple interneurons around spindle troughs might contribute to precise tuning of rapidly successive excitation and inhibition (Isaacson and Scanziani, 2011; Wehr and Zador, 2003). Similarly, alternating activation of interneurons and pyramidal cells at high-gamma and ripple frequencies has been reported from human neocortex in vitro (Molnár et al., 2008) and rodent in vivo preparations (Stark et al., 2014, 2015). Monosynaptic connections linking specific subpopulations of FS interneurons to pyramidal cells and to other FS interneurons could provide cooperative feedback and reciprocal inhibition, in combination with extracortical excitatory drive, forming the synaptic background of the spindle-trough-centered, high-frequency events.

In-depth interpretation of similarities in spectral and phase properties of firing and local field potentials juxtacellular to spindle ripple and spindle high-gamma cells is complex due to known and undefined factors linking single-cell membrane and firing properties to population events (Buzsáki et al., 2012). In vivo intracellularly recorded individual FS cells were suggested to regulate fast network oscillations in phase with single interneuron activity (Jones et al., 2000). The firing of an individual spindle ripple cell might contribute to the generation/entrainment of ripple and high-gamma band population oscillations in the neighborhood of the cell, and it is also plausible that a single spindle high-gamma cell is involved in the generation/entrainment of high-gamma band population oscillations in its vicinity. However, based on the relatively moderate frequency of spikes in individual FS cells phase coupled to ongoing local field rhythms, we speculate that single FS cells might not be sufficient for the entrainment of populations even of restricted cortical volumes, and such entrainment is likely to require concerted action of several interneurons of similarly tuned FS cell functional subgroups, as put forward by earlier studies (Cardin et al., 2009; Galarreta and Hestrin, 1999; Gibson et al., 1999; Isaacson and Scanziani, 2011; Swadlow et al., 1998). Cooperation of distributed and synchronized networks of FS cell subgroups, i.e., coherent groups of several FS cells with similar spectral properties in their output, could explain why we detect high-gamma and ripple band oscillations in spindle cycles with and without spikes juxtacellular to particular members of FS cell subgroups.

Simultaneously ongoing or nested oscillations are characteristic of the operation of cortical networks and were suggested to be instrumental in several forms of higher-order cognitive processes (Buzsáki and Draguhn, 2004; McBain and Fisahn, 2001; Salinas and Sejnowski, 2001; Singer and Gray, 1995; Steriade, 2000). To our knowledge, the precision revealed here at which the trough of high-gamma and the peak of ripple band oscillations are timed to the trough of the ongoing spindle is unprecedented in phase-phase coupling between oscillations of different frequencies. Sharply tuned firing of spindle ripple and spindle high-gamma cells riding on subsequent peaks of the juxtacellularly recorded field potential could maintain population synchronization through fast

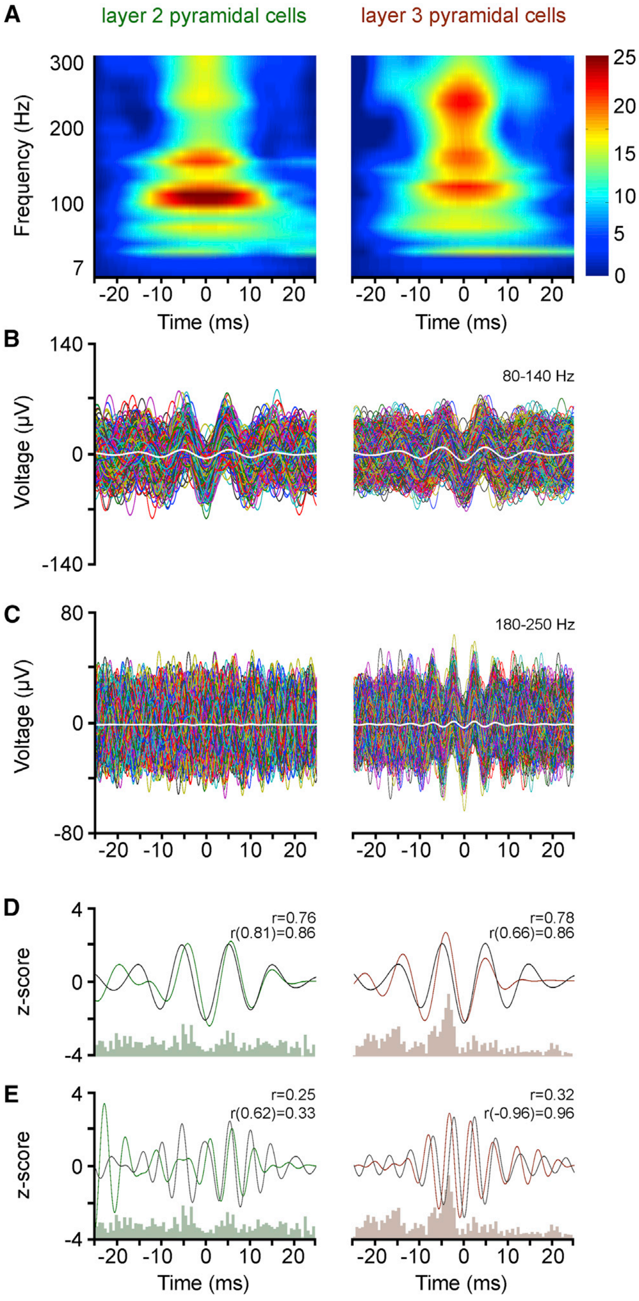

Figure 6. High-Frequency Network Events at the Trough of Spindle Oscillations Recorded Juxtacellularly around Identified Pyramidal Cells

(A) Average wavelet spectra constructed from spindle troughs (timed at 0 ms) during which juxtacellular spikes were absent in pyramidal cells. Both ripple band and high-gamma activity emerge around spindle troughs in recordings juxtacellular to layer III pyramidal cells, but activity predominantly in the high-gamma range is characteristic of recordings juxtacellular to layer II pyramidal cells.

(B and C) Consecutive individual traces filtered 80–140 Hz (B) and 180–250 Hz (C) aligned according to spindle troughs (0 ms). Colored traces show all spindle troughs without spikes available from a representative cell from layer II and III pyramidal cells (same cell for B and C); averages of layer II and III pyramids are shown in white. Oscillations in the high-gamma range appear in both pyramidal cell populations, and the trough of high-gamma oscillations coincides with the spindle trough. Ripple oscillations are predominant in layer III pyramidal cells and the trough of ripple oscillations is at the trough of spindles.

(D and E) Foreground: correlation between the firing (green and brown; kernel-smoothed firing frequency distributions bandpass filtered at 80–140 Hz, D, and 180–250 Hz, E) and network activity (black; z-scored averages shown in B and C) recorded juxtacellularly to pyramidal cells (r, Pearson correlation coefficients). Background: average firing frequency distribution histograms of pyramidal cells.

$GABA_A$ receptor-mediated output combined with pyramidal cell recruitment for a couple of subsequent cycles at these frequencies (Stark et al., 2014, 2015). However, the onset and offset of high-gamma and ripple band events prior to and after the trough of spindles speculatively require additional thalamocortical mechanisms, suggested to recruit different interneurons with variable latencies (Cruikshank et al., 2010; Swadlow, 2003) and presumably intracortical processes, like autaptic basket cell connections and reciprocal synaptic coupling within and outside the population of FS cells (Bacci and Huguenard, 2006; Cruikshank et al., 2010). Neuron populations of transient neocortical high-frequency network events around spindle troughs might serve as successive temporal references for sorting and consolidating information arriving through long-range connections from other brain areas (Peyrache et al., 2011). This process might be particularly effective for temporally structured epochs such as hippocampal ripples, which could be time mapped and sorted according to neocortical rhythms of similar frequency through a scenario repeated at intervals generated by the undergoing spindle oscillations.

EXPERIMENTAL PROCEDURES

All experimental protocols and procedures were performed according to the European Communities Council Directives of 1986 (86/609/EEC) and 2003 (2003/65/CE) for animal research and were approved by the Ethics Committee of the University of Szeged. Male rats (300–500 g, RRID: RGD_2312511) were pre-anesthetized with a ketamine/xylasine mixture (70 mg/8 mg/kg) and placed into a stereotaxic frame. Unilateral craniotomies of ~2.3 mm diameter were performed above left or right parietal (anteroposterior [AP], –3.8–4.2 mm; mediolateral [ML], 2.2–2.5 mm; 31 animals) or secondary motor cortex (AP, +1.7–+2.2 mm; ML, 0.8–1.0 mm; 4 animals) under isoflurane anesthesia (0.6%–0.8% at the first hour and 1.2%–1.5% later) and constant monitoring of body temperature (37°C). In order to protect the area of surgical interventions from active tissue regrowth, we applied 0.01% Mitomicin (Sigma) to the intact dura mater up to 10 min and covered the site of craniotomy with drops of 1.5%–2% agar. A holding platform for the pipette microdrive assembly, including a chamber (inner diameter [ID], 4 mm; height [H], 2 mm), was attached to the skull above the agar-coated hole with phosphate cement and further enforced with acrylic cement (Korshunov, 1995, 2006). The chamber was filled with agar and covered with a cup to avoid drying. Reference and grounding electrodes soldered to male 0.05 inch pins (50 μm NiCr formwar insulation and 150 μm PtIr bare wires, respectively) were implanted onto the contralateral hemisphere.

Post-surgical recovery was combined with adaptation to the experimental recording situation 4–5 consecutive days following the operation. Animals were moved to a recording theater (40 × 40 × 27 cm, W × D × H, respectively) with bedding on its floor and ad libitum water and food supply. The recording theater was placed in an acoustically isolated and electrically shielded room (150 × 150 × 220 cm, W × D × H, respectively). The animals spent 2 × 4–5 hr per day in the experimental theater to develop a daytime routine. When moving the animals in a shuttle box in between their home cages and the experimental theater, the area of surgery was cleaned and filled with agar every day. Sessions of drug-free installation and usage of the microdrive

assembly (without electrodes) imitating real experiments began on the third day of training. This included episodes in which the experimenter approached sleeping animals and made small steps with microdrive manually (resolution 350 mm/turn). When animals learned to tolerate the periodic short presence of experimenters near the experimental cage without movement in their sleep while manipulating the microdrives (on the 5th–6th post-operative day), juxtacellular experiments were started. Installation of the experimental assembly is shown in Movie S1.

Thick-walled borosilicate (A-M Systems, Sutter, outer diameter [OD], 1.0 mm; ID, 58 mm) capillaries were used to produce micropipettes of alternating taper morphology (taper length 5–10 mm) capable of penetrating the dura mater with electrode tips suitable for juxtacellular recordings. Pipettes were filled with 0.5 M NaCl containing 2% neurobiotin (Vector), had resistances between 7 and 12 MΩ, and were advanced by microdrives, as described previously (Korshunov, 1995). Juxtacellular labeling (Pinault, 1996) was performed by injecting positive current pulses (0.5–18 nAm; 150–250 ms). Sharpened tungsten electrodes (1.0–1.5 MΩ, FHC) were advanced by a second, ultraminiature microdrive (Korshunov, 2006) at an angle of ~7° to approach the juxtacellular pipette to achieve a recording distance of 250–500 μm in n = 15 animals. Wideband signals from preamplifiers (ELC-MINI-DIFF-2, NPI Electronic; unity gain) were amplified (×1,000), filtered (1 Hz–5 kHz), and processed (CED1403Power3) for offline analysis in Spike2 and custom-made scripts in MATLAB 2013a. In n = 3 additional animals, the tungsten electrode was advanced to the dorsal CA1 (AP, 3.0–3.5; ML, 1.8–2.3) above the pyramid layer to record sharp-wave ripples in parallel with cortical spindles.

A camera (640 × 480 resolution) was mounted above the experimental theater to monitor the behavior. Behavioral states were defined offline by identifying awake and sleep epochs based on video recordings. Active awake periods were defined based on exploration of the recording theater, self-cleaning behavior, or consumption of food and short awake states in resting position before the sleep periods when animals occupied the preferred corner. Sleep was characterized by a curled-up or ball posture with eyes closed. In addition, we used a headstage with LEDs and an accelerometer that generated voltage deflections during movements in n = 6 animals. The SWS was characterized by slow-wave activity with occasional k-complexes or slow waves/k-complexes interchanging with spindle episodes (Figures 1 and S1). The REM sleep was characterized by rhythmic activity at the theta band (6–12 Hz) in the cortical local field potential.

After transcardial perfusion (4% paraformaldehyde, 15% v/v saturated picric acid, and 0.05% glutaraldehyde in 0.1 M phosphate buffer [PB]), brains were postfixed overnight and stored in 0.1 M PB with 0.05% sodium azide (PB-Az) at 4°C. Coronal sections (50–70 μm; Leica VT 1000S vibratome) were either permeabilized in Tris-buffered saline (TBS) with 0.1% Triton X-100 (Tx) or subjected to freeze-thaw over liquid nitrogen after cryoprotecting in 20% sucrose. The sections were incubated in Streptavidin-conjugated AlexaFluor 488 (1:1,000; Molecular Probes) in TBS or TBS-Tx for 2 hr at room temperature (RT) or overnight at 4°C. Parvalbumin immunoreactivity of neurobiotin-labeled cells was tested using goat anti-parvalbumin (1:5,000; Swant) and mouse-anti parvalbumin (1:2,000; Swant) primary antibodies, and co-localization was checked with an Olympus FV1000 confocal microscope.

For analysis and 3D reconstructions, sections were incubated in 1:100 avidin-biotinylated peroxidase complex (ABC; Vectastain ABC Elite kit, Vector Laboratories) in TBS for 1–3 days at 4°C and in a 0.5 mg/mL diaminobenzidine (DAB; Electron Microscopy Sciences) solution containing glucose, ammonium chloride, and nickel ammonium sulfate in 0.1 M PB for 15 min. After 15 min, 0.5% glucose oxidase was added to generate H_2O_2 for the oxidation of DAB by horseradish peroxidase. After 40 min, sections were treated with osmium tetroxide in 0.1 M PB for 1 hr and uranyl acetate in distilled water for 25 min. After dehydration (50% –100% ethanol), sections were mounted in resin (Durcupan; Sigma-Aldrich). Three-dimensional light microscopic reconstructions were performed using Neurolucida 8.23 (MBF Bioscience) with 100× objective.

Data analysis was performed using routines written in MATLAB 2013a (MathWorks). Wideband recordings (sampled at 40 Khz) from juxtaposition were downsampled by 8, low-pass filtered by forward-backward-zero-phase finite impulse response (FIR) filters (4–20 Hz and 4–120 Hz), and used for automatic spindle trough detection (Gardner et al., 2013) with Bayesian despiking algorithm (Zanos et al., 2011) and manual post-correction. We applied CircStat toolbox for MATLAB for analyzing spindle phase relatedness. To decide if there was any phase preference in the spindle wave, we calculated the resultant mean vector's direction and length and the circular variance for the binned data. The population (n = 21) MVD and the dispersion around the mean (DAM) were calculated for the mean vectors of FS cells. Then a unimodal von Mises distribution was generated with parameters μ = MVD and κ = 1/DAM. The same number (n = 21) of samples had been drawn from this distribution, and then it was compared to the original distribution of MVDs with Kuiper's two-sample test. The Kuiper's test hypothesis was rejected if $p > 0.05$. This process was iterated 1,000 times. Kernel smoothing was applied with a significance level of $p > 0.99$ (one-sample Kolmogorov-Smirnov test) to estimate the probability density function (PDF) of firing activity of each FS cell and the PDF of the cumulative firing of layer II and III pyramidal cells in time windows around spindle troughs (± 25 ms) and we searched for the local maxima of this estimated density function for determining the number and timing of peaks. For correlations between the firing of neurons and juxtacellularly recorded local field potentials, the resultant PDFs were filtered with a bandpass FIR filter between 80 and 140 Hz and 180 and 250 Hz, and then Pearson correlation coefficient was calculated between filtered PDF signals and the averaged juxtacellularly recorded local field potentials filtered between 80 and 140 Hz (spindle high gamma) and 180 and 250 Hz (spindle ripple). For calculating p values, we used surrogate data testing with random shuffle generation of 1,000 surrogate data by random permutations of the original firing distributions for each cell group.

Peri-spindle trough time histograms (PSTHs) were constructed using a bin width of 0.78 ms for all cell classes. The timing of action potentials relative to spindle troughs in SWS was based on >250 troughs for each FS cell (n = 21; 993.86 ± 512.5 troughs) and >500 troughs for pyramidal cells (n = 28, 1,122 ± 514 troughs). A grand average of the mean PSTHs was calculated by pooling individual PSTHs in a given cell group. Phase-amplitude coupling analysis was based on wide band signals recorded juxtacellular to pyramidal cells, which were bandpass filtered between 6 and 20 Hz for spindle phase estimation with Hilbert transformation, and 75–130 Hz and 180–250 Hz for the high-gamma and ripple band, respectively, for amplitude estimation. High-gamma and ripple amplitudes were averaged degree by degree, covering all phases of spindle cycles. We divided these values with the sum of all values to get a normalized average amplitude for a cell. To describe the strength of the phase-amplitude coupling, the modulation index (MI) was calculated based on Kullback-Leibler divergence. A surrogate test was performed on the dataset for significance levels: amplitude values were randomly shuffled multiple times, followed by the calculation of the MI from the shuffled data. The distribution of the MI of the randomized series showed that the significance threshold was below our experimental MI results. Time-frequency analysis of spindle, high-gamma, and ripple band components was used through continuous Morlet-wavelet transformation. Wavelet power was calculated as squared absolute values of wavelet coefficients, and wavelet power was normalized (using its own z score for the analysis of the spindle and using a baseline for the analysis of the near-trough episodes) to remove the 1/f nature of the spectral power.

Group results are given as mean ± SEM with individual mean data plotted unless stated otherwise. Statistical analysis was performed using MATLAB or SPSS 15.0 considering $p < 0.05$ as statistically significant.

SUPPLEMENTAL INFORMATION

Supplemental Information includes five figures, one table, and one movie and can be found with this article online at http://dx.doi.org/10.1016/j.neuron.2016.09.032.

AUTHOR CONTRIBUTIONS

R.G.A. conducted electrophysiological recordings; V.S. performed light microscopic processing, reconstruction, and immunohistochemistry; S.B., R.G.A., and G.T. analyzed data; and G.T. and R.G.A. wrote the manuscript.

ACKNOWLEDGMENTS

The authors thank L. Acsády, A. Berényi, G. Buzsáki, and L. Menendez de la Prida for comments on the manuscript. This work was supported by the ERC Interimpact project (G.T.), the Hungarian Academy of Sciences (G.T.), the Hungarian National Office for Research and Technology GINOP-2.3.2-15-2016-00018, and the National Brain Research Program, Hungary (G.T.).

Received: March 9, 2016
Revised: June 25, 2016
Accepted: August 31, 2016
Published: October 13, 2016

REFERENCES

Atallah, B.V., and Scanziani, M. (2009). Instantaneous modulation of gamma oscillation frequency by balancing excitation with inhibition. Neuron *62*, 566–577.

Bacci, A., and Huguenard, J.R. (2006). Enhancement of spike-timing precision by autaptic transmission in neocortical inhibitory interneurons. Neuron *49*, 119–130.

Beierlein, M., Gibson, J.R., and Connors, B.W. (2003). Two dynamically distinct inhibitory networks in layer 4 of the neocortex. J. Neurophysiol. *90*, 2987–3000.

Born, J., Rasch, B., and Gais, S. (2006). Sleep to remember. Neuroscientist *12*, 410–424.

Burgalossi, A., Herfst, L., von Heimendahl, M., Förste, H., Haskic, K., Schmidt, M., and Brecht, M. (2011). Microcircuits of functionally identified neurons in the rat medial entorhinal cortex. Neuron *70*, 773–786.

Buzsáki, G. (1996). The hippocampo-neocortical dialogue. Cereb. Cortex *6*, 81–92.

Buzsáki, G., and Draguhn, A. (2004). Neuronal oscillations in cortical networks. Science *304*, 1926–1929.

Buzsáki, G., Horváth, Z., Urioste, R., Hetke, J., and Wise, K. (1992). High-frequency network oscillation in the hippocampus. Science *256*, 1025–1027.

Buzsáki, G., Anastassiou, C.A., and Koch, C. (2012). The origin of extracellular fields and currents—EEG, ECoG, LFP and spikes. Nat. Rev. Neurosci. *13*, 407–420.

Cardin, J.A., Carlén, M., Meletis, K., Knoblich, U., Zhang, F., Deisseroth, K., Tsai, L.-H., and Moore, C.I. (2009). Driving fast-spiking cells induces gamma rhythm and controls sensory responses. Nature *459*, 663–667.

Clemens, Z., Mölle, M., Eross, L., Jakus, R., Rásonyi, G., Halász, P., and Born, J. (2011). Fine-tuned coupling between human parahippocampal ripples and sleep spindles. Eur. J. Neurosci. *33*, 511–520.

Contreras, D., Destexhe, A., Sejnowski, T.J., and Steriade, M. (1997). Spatiotemporal patterns of spindle oscillations in cortex and thalamus. J. Neurosci. *17*, 1179–1196.

Cruikshank, S.J., Lewis, T.J., and Connors, B.W. (2007). Synaptic basis for intense thalamocortical activation of feedforward inhibitory cells in neocortex. Nat. Neurosci. *10*, 462–468.

Cruikshank, S.J., Urabe, H., Nurmikko, A.V., and Connors, B.W. (2010). Pathway-specific feedforward circuits between thalamus and neocortex revealed by selective optical stimulation of axons. Neuron *65*, 230–245.

Csicsvari, J., Hirase, H., Czurkó, A., Mamiya, A., and Buzsáki, G. (1999). Oscillatory coupling of hippocampal pyramidal cells and interneurons in the behaving Rat. J. Neurosci. *19*, 274–287.

Diekelmann, S., and Born, J. (2010). The memory function of sleep. Nat. Rev. Neurosci. *11*, 114–126.

Douglas, R.J., and Martin, K.A.C. (2004). Neuronal circuits of the neocortex. Annu. Rev. Neurosci. *27*, 419–451.

Einevoll, G.T., Kayser, C., Logothetis, N.K., and Panzeri, S. (2013). Modelling and analysis of local field potentials for studying the function of cortical circuits. Nat. Rev. Neurosci. *14*, 770–785.

Engel, A.K., Fries, P., and Singer, W. (2001). Dynamic predictions: oscillations and synchrony in top-down processing. Nat. Rev. Neurosci. *2*, 704–716.

English, D.F., Peyrache, A., Stark, E., Roux, L., Vallentin, D., Long, M.A., and Buzsáki, G. (2014). Excitation and inhibition compete to control spiking during hippocampal ripples: intracellular study in behaving mice. J. Neurosci. *34*, 16509–16517.

Fisahn, A., Pike, F.G., Buhl, E.H., and Paulsen, O. (1998). Cholinergic induction of network oscillations at 40 Hz in the hippocampus in vitro. Nature *394*, 186–189.

Galarreta, M., and Hestrin, S. (1999). A network of fast-spiking cells in the neocortex connected by electrical synapses. Nature *402*, 72–75.

Gardner, R.J., Hughes, S.W., and Jones, M.W. (2013). Differential spike timing and phase dynamics of reticular thalamic and prefrontal cortical neuronal populations during sleep spindles. J. Neurosci. *33*, 18469–18480.

Gibson, J.R., Beierlein, M., and Connors, B.W. (1999). Two networks of electrically coupled inhibitory neurons in neocortex. Nature *402*, 75–79.

Hartwich, K., Pollak, T., and Klausberger, T. (2009). Distinct firing patterns of identified basket and dendrite-targeting interneurons in the prefrontal cortex during hippocampal theta and local spindle oscillations. J. Neurosci. *29*, 9563–9574.

Hasenstaub, A., Shu, Y., Haider, B., Kraushaar, U., Duque, A., and McCormick, D.A. (2005). Inhibitory postsynaptic potentials carry synchronized frequency information in active cortical networks. Neuron *47*, 423–435.

Huguenard, J.R., and McCormick, D.A. (2007). Thalamic synchrony and dynamic regulation of global forebrain oscillations. Trends Neurosci. *30*, 350–356.

Isaacson, J.S., and Scanziani, M. (2011). How inhibition shapes cortical activity. Neuron *72*, 231–243.

Jones, M.S., MacDonald, K.D., Choi, B., Dudek, F.E., and Barth, D.S. (2000). Intracellular correlates of fast (>200 Hz) electrical oscillations in rat somatosensory cortex. J. Neurophysiol. *84*, 1505–1518.

Kandel, A., and Buzsáki, G. (1997). Cellular-synaptic generation of sleep spindles, spike-and-wave discharges, and evoked thalamocortical responses in the neocortex of the rat. J. Neurosci. *17*, 6783–6797.

Klausberger, T., and Somogyi, P. (2008). Neuronal diversity and temporal dynamics: the unity of hippocampal circuit operations. Science *321*, 53–57.

Korshunov, V.A. (1995). Miniature microdrive for extracellular recording of neuronal activity in freely moving animals. J. Neurosci. Methods *57*, 77–80.

Korshunov, V.A. (2006). Miniature microdrive-headstage assembly for extracellular recording of neuronal activity with high-impedance electrodes in freely moving mice. J. Neurosci. Methods *158*, 179–185.

McBain, C.J., and Fisahn, A. (2001). Interneurons unbound. Nat. Rev. Neurosci. *2*, 11–23.

McClelland, J.L., McNaughton, B.L., and O'Reilly, R.C. (1995). Why there are complementary learning systems in the hippocampus and neocortex: insights from the successes and failures of connectionist models of learning and memory. Psychol. Rev. *102*, 419–457.

Meyer, A.H., Katona, I., Blatow, M., Rozov, A., and Monyer, H. (2002). In vivo labeling of parvalbumin-positive interneurons and analysis of electrical coupling in identified neurons. J. Neurosci. *22*, 7055–7064.

Molnár, G., Oláh, S., Komlósi, G., Füle, M., Szabadics, J., Varga, C., Barzó, P., and Tamás, G. (2008). Complex events initiated by individual spikes in the human cerebral cortex. PLoS Biol. *6*, e222.

Patel, J., Schomburg, E.W., Berényi, A., Fujisawa, S., and Buzsáki, G. (2013). Local generation and propagation of ripples along the septotemporal axis of the hippocampus. J. Neurosci. *33*, 17029–17041.

Peyrache, A., Battaglia, F.P., and Destexhe, A. (2011). Inhibition recruitment in prefrontal cortex during sleep spindles and gating of hippocampal inputs. Proc. Natl. Acad. Sci. USA *108*, 17207–17212.

Pinault, D. (1996). A novel single-cell staining procedure performed in vivo under electrophysiological control: morpho-functional features of juxtacellularly

labeled thalamic cells and other central neurons with biocytin or Neurobiotin. J. Neurosci. Methods *65*, 113–136.

Puig, M.V., Ushimaru, M., and Kawaguchi, Y. (2008). Two distinct activity patterns of fast-spiking interneurons during neocortical UP states. Proc. Natl. Acad. Sci. USA *105*, 8428–8433.

Rasch, B., and Born, J. (2013). About sleep's role in memory. Physiol. Rev. *93*, 681–766.

Royer, S., Sirota, A., Patel, J., and Buzsáki, G. (2010). Distinct representations and theta dynamics in dorsal and ventral hippocampus. J. Neurosci. *30*, 1777–1787.

Salinas, E., and Sejnowski, T.J. (2001). Correlated neuronal activity and the flow of neural information. Nat. Rev. Neurosci. *2*, 539–550.

Singer, W., and Gray, C.M. (1995). Visual feature integration and the temporal correlation hypothesis. Annu. Rev. Neurosci. *18*, 555–586.

Sohal, V.S., Zhang, F., Yizhar, O., and Deisseroth, K. (2009). Parvalbumin neurons and gamma rhythms enhance cortical circuit performance. Nature *459*, 698–702.

Staresina, B.P., Bergmann, T.O., Bonnefond, M., van der Meij, R., Jensen, O., Deuker, L., Elger, C.E., Axmacher, N., and Fell, J. (2015). Hierarchical nesting of slow oscillations, spindles and ripples in the human hippocampus during sleep. Nat. Neurosci. *18*, 1679–1686.

Stark, E., Roux, L., Eichler, R., Senzai, Y., Royer, S., and Buzsáki, G. (2014). Pyramidal cell-interneuron interactions underlie hippocampal ripple oscillations. Neuron *83*, 467–480.

Stark, E., Roux, L., Eichler, R., and Buzsáki, G. (2015). Local generation of multineuronal spike sequences in the hippocampal CA1 region. Proc. Natl. Acad. Sci. USA *112*, 10521–10526.

Steriade, M. (2000). Corticothalamic resonance, states of vigilance and mentation. Neuroscience *101*, 243–276.

Swadlow, H.A. (2003). Fast-spike interneurons and feedforward inhibition in awake sensory neocortex. Cereb. Cortex *13*, 25–32.

Swadlow, H.A., Beloozerova, I.N., and Sirota, M.G. (1998). Sharp, local synchrony among putative feed-forward inhibitory interneurons of rabbit somatosensory cortex. J. Neurophysiol. *79*, 567–582.

Terrier, G., and Gottesmann, C.L. (1978). Study of cortical spindles during sleep in the rat. Brain Res. Bull. *3*, 701–706.

Tiesinga, P., and Sejnowski, T.J. (2009). Cortical enlightenment: are attentional gamma oscillations driven by ING or PING? Neuron *63*, 727–732.

Valero, M., Cid, E., Averkin, R.G., Aguilar, J., Sanchez-Aguilera, A., Viney, T.J., Gomez-Dominguez, D., Bellistri, E., and de la Prida, L.M. (2015). Determinants of different deep and superficial CA1 pyramidal cell dynamics during sharp-wave ripples. Nat. Neurosci. *18*, 1281–1290.

Varga, C., Oijala, M., Lish, J., Szabo, G.G., Bezaire, M., Marchionni, I., Golshani, P., and Soltesz, I. (2014). Functional fission of parvalbumin interneuron classes during fast network events. eLife *3*, http://dx.doi.org/10.7554/eLife.04006.

Wehr, M., and Zador, A.M. (2003). Balanced inhibition underlies tuning and sharpens spike timing in auditory cortex. Nature *426*, 442–446.

Wilson, M.A., and McNaughton, B.L. (1994). Reactivation of hippocampal ensemble memories during sleep. Science *265*, 676–679.

Zanos, T.P., Mineault, P.J., and Pack, C.C. (2011). Removal of spurious correlations between spikes and local field potentials. J. Neurophysiol. *105*, 474–486.

Neuron, Volume *92*

Supplemental Information

Identified Cellular Correlates of Neocortical Ripple and High-Gamma Oscillations during Spindles of Natural Sleep

Robert G. Averkin, Viktor Szemenyei, Sándor Bordé, and Gábor Tamás

Supplemental Information

1. Inventory of Supplemental Information

Figure S1 illustrates recording quality and duration of our methodologically demanding recordings and overall data supplemental Figure 1.

Figure S2 is essential to further explain Figures 2-3 by presenting the stability of spindle ripple and spindle high gamma cell behavior over the course of spindle showing the lack of phase precession or phase lag as the spindle progresses, and illustrates illustrates FS cell heterogeneity, clustering, spike triggered firing latencies and the distribution of firing relative to peaks of activity.

Figure S3 shows that FS cell groups are not a product of spindle heterogeneity.

Figure S4 extends Figure 4 in showing similar phenomena in spindle cycles with spikes (as opposed to without spikes in Figure 4).

Figure S5 illustrates the variability of firing distribution of layer II and III pyramidal cells.

Table S1 presents data measured for FS cell subgroups in detail.

Video S1, related to Figure 1 is a step by step demonstration of our drug free in vivo recordings.

2. Supplemental Items

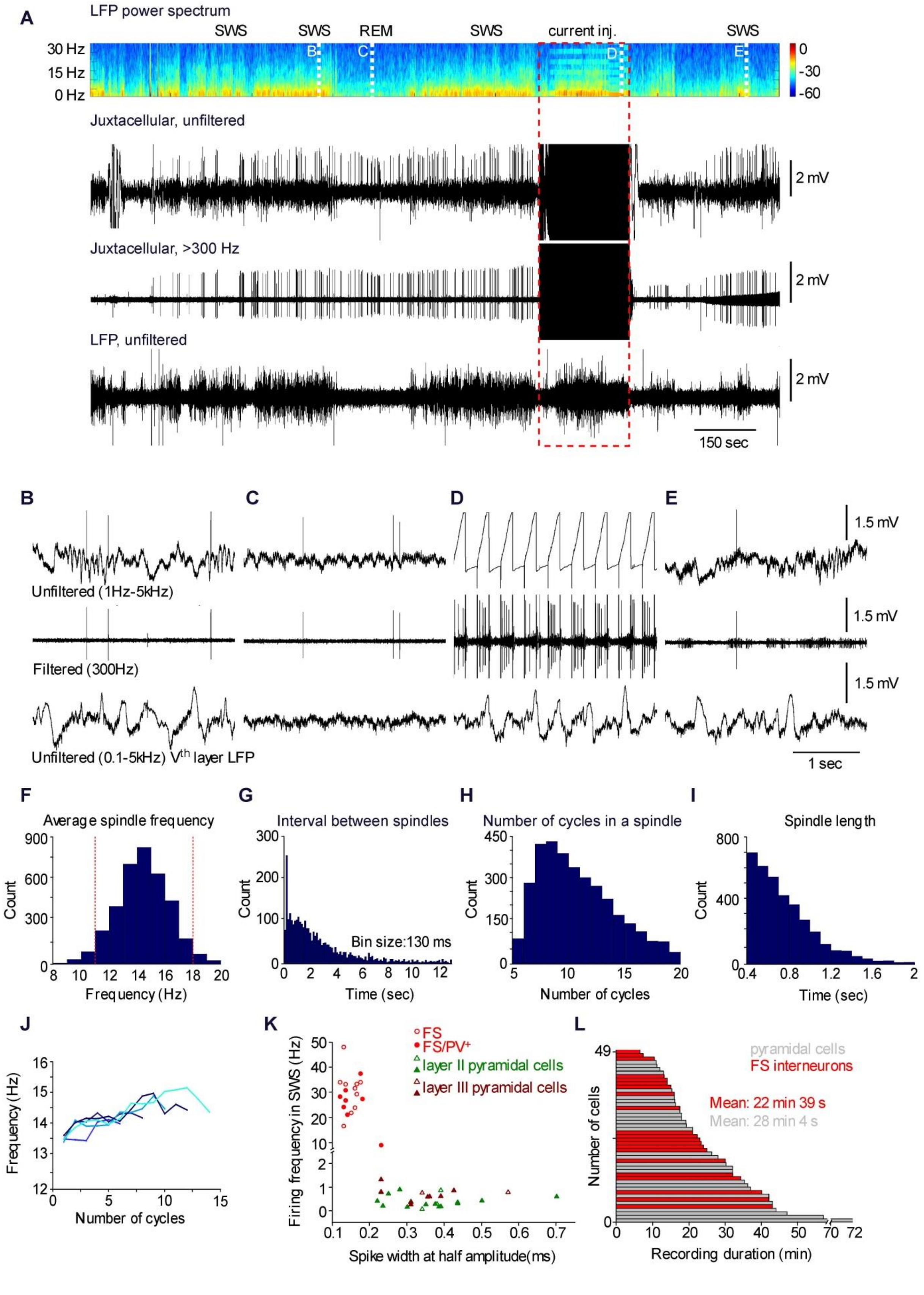

Figure S1, related to Figure 1. (**A**) Example of raw traces recorded in juxtacellular position to 2 layer II pyramidal cell with simultaneous local field potential (LFP) recording in layer V. Top panel, Power spectrum of the LFP. Top and middle trace, Wideband (top) and high pass filtered (middle) juxtacellular recording of the

layer II pyramidal cell activity. Bottom trace, Wideband raw trace of the layer V LFP. (**B-E**) Episodes of recordings presented on panel **A** shown at a detailed timescale during slow wave sleep (SWS, **B**, **E**), rapid eye movement sleep (REM, **C**) and positive current injection (current inj, **D**). (**F-J**) Distribution of internal spindle frequency (**F**), intervals separating successive spindles (**G**), the number of cycles per spindle (H) and of the length of spindles (**I**) in our overall dataset. (**J**) Instantaneous frequency of spindle cycles depending on the length of spindles. (**K**) Segregation of FS cells and pyramidal cells based on spike width and firing frequencies during SWS. (**L**) Length of continuous recordings at juxtacellular position to pyramidal cells and FS cells in freely behaving rats.

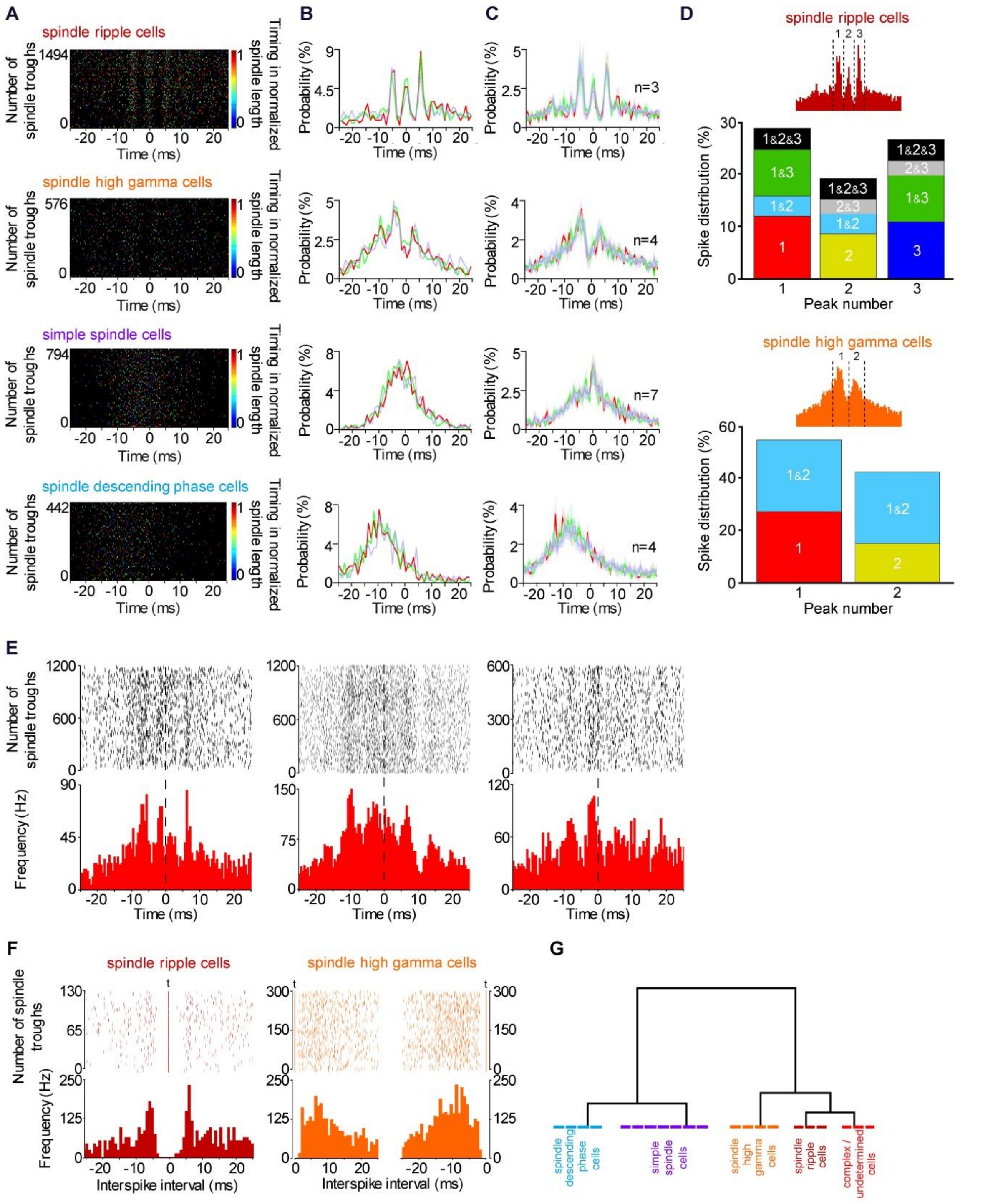

Figure S2, related to Figure 2 and 3. (**A**) Distribution of action potentials of representative examples from four groups of fast spiking cells (top to bottom: spindle ripple cells, spindle high gamma cells, simple spindle cells, spindle descending phase cells) color coded according to their timing relative to the normalized length of the actual ongoing spindle. The mixture of colors throughout the scattergram indicate the absence of phase precession or phase lag of spikes relative to spindle troughs. (**B**) High temporal resolution firing probability of the cells shown on **A** during the first (blue), second (green) and last third of spindle cycles (red). (**C**) Average high temporal resolution firing probability distributions of the four fast spiking cell populations during the first (blue), second (green) and last third of spindle cycles (red). Shadowed areas correspond to SEM. (**D**) Bottom, Examination of firing during single spindle troughs revealed that individual spindle high gamma cells fired

during the first (1) second (2) or both (1 & 2) peaks of overall activity (inset). Top, Single spindle ripple cells could be active during the first (1), second (2) or third (3) peak only, during two peaks (1 &2, 1 & 3, 2 & 3) and during all three peaks (1 & 2 & 3) of overall activity (inset). (**E**) Spindle trough related FS cells with complex high frequency timing of firing around spindle troughs (0 ms). These cells could not be classified as spindle ripple, spindle high gamma or simple spindle cells. (**F**) Spike triggered analysis of interspike intervals relative to action potentials of particular peaks of spindle-high gamma cells or spindle-ripple cells. Left, Interspike interval distribution of action potentials triggered (t) on spikes of the central peak of activity in a spindle ripple cell. Right, Latencies of spikes relative to action potentials in a spindle high gamma cell. The left and right panels show distributions triggered (t) relative to action potentials of the first and second peak of activity, respectively. (**G**) Hierarchical clustering using Ward's method based on the number of peaks in firing probability in ± 12.5 ms and ± 6.25 ms time windows centered at the trough of spindles resulted in five cell clusters corresponding to spindle ripple, spindle high gamma, simple spindle, spindle descending phase and complex / undetermined cells. Clustering was based on spindle phase relative to the trough.

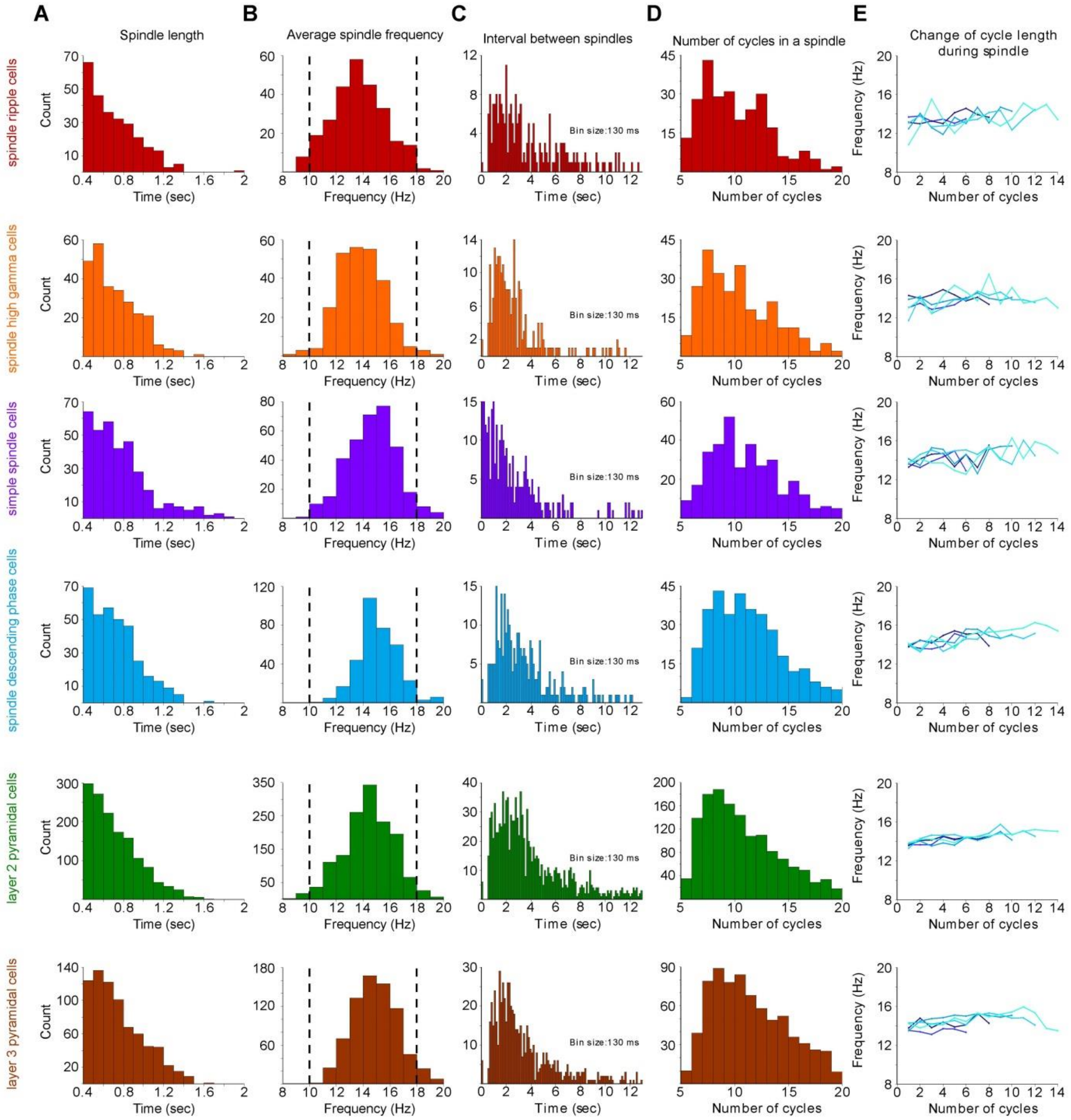

Figure S3, , related to Figure 2 and 3. Distribution of internal spindle length (**A**) average spindle frequency (**B**), intervals separating successive spindles (**C**), the number of cycles per spindle (**D**) in groups of FS cells and pyramidal cells. (**E**) Instantaneous frequency of spindle cycles depending on the length of spindles.

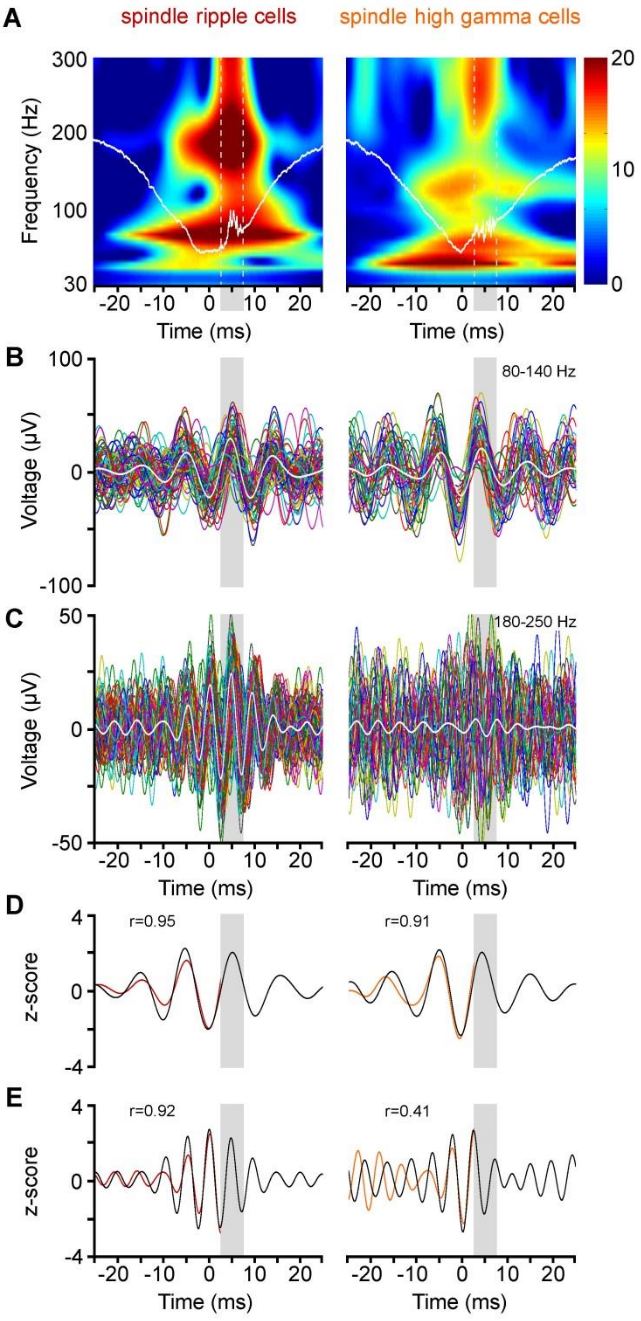

Figure S4, related to Figure 4. Presence of gamma and ripple band oscillatory field activity in spindle cycles with juxtacellularly detected firing. (**A**) Average wavelet spectra constructed from spindle troughs (timed at 0 ms) during which juxtacellular spikes were timed 2.5 to 7.5 ms (dashed lines and gray band) after the spindle trough in functional subgroups of FS cells. Both ripple-band and high gamma activity emerge around spindle troughs and prior to the spikes in recordings juxtacellular to spindle ripple cells, but activity predominantly in the high gamma range is characteristic to recordings juxtacellular to spindle high gamma cells. (**B-C**) Consecutive individual traces filtered 80-140 Hz (B) and 180-250 Hz (C) aligned according to spindle troughs (0 ms). Colored traces show all spindle troughs with spikes in the time window 2.5 to 7.5 ms after the trough (grey bands) available from a representative cell from each FS interneuron subgroup (same cell for B and C), averages of subgroups are shown in white. Oscillations in the high gamma range appear in both FS cell subgroups and the through of high gamma oscillations coincide with the spindle trough. Ripple oscillations are predominant in spindle ripple cells and the peak of ripple oscillations is at the trough of spindles. (**D, E**) FS cell subgroup dependent correlation between local field activity in spindle cycles with spikes (burgundy, orange; z-scored averages shown on panels B and C) and without spikes (black) bandpass filtered at 80-140 Hz and 180-250 Hz

recorded juxtacellularly to FS interneurons (r, Pearson correlation coefficients). Using the common time reference of spindle troughs, correlations were calculated for the time window of -25 to +2.5 ms relative to the trough between filtered and z-scored averages of local field potentials derived from spindle cycles with and without spikes.

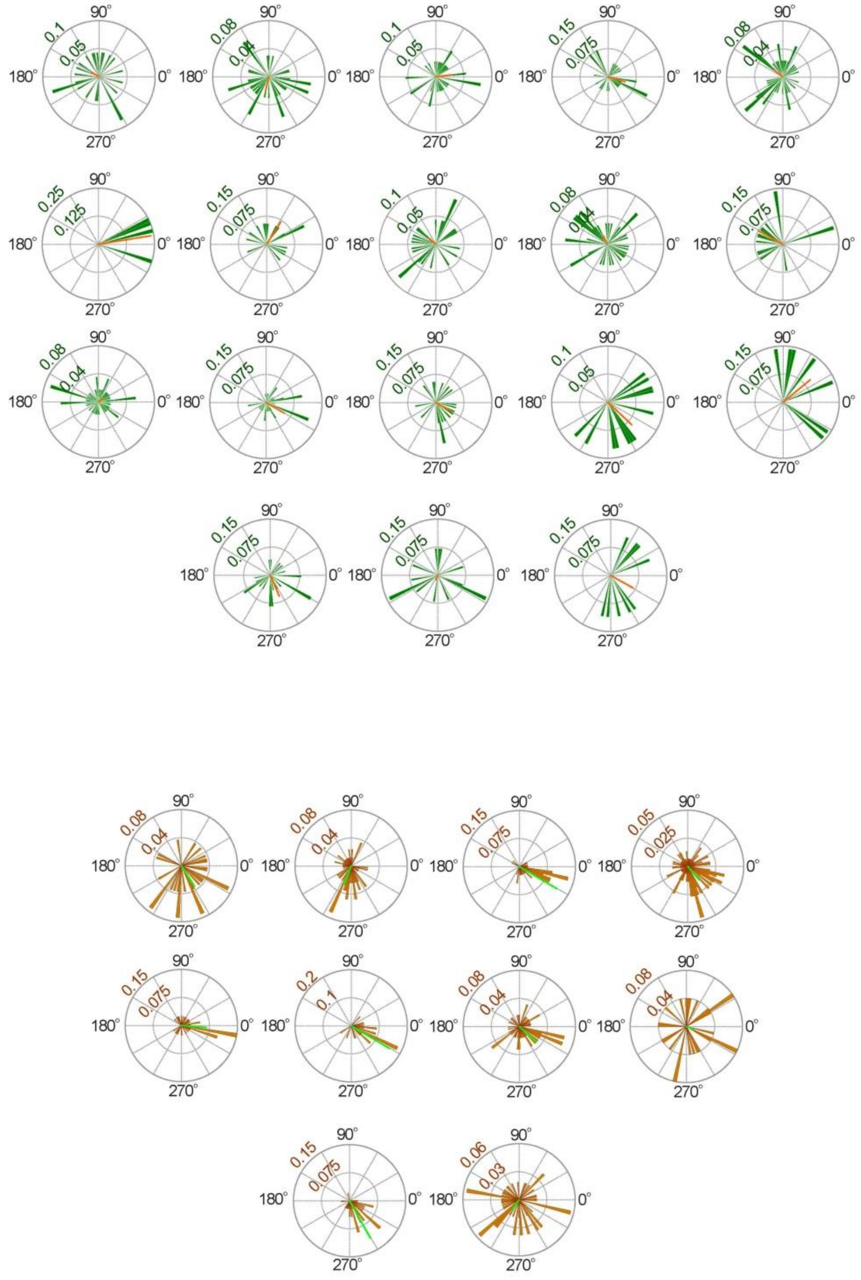

Figure S5, related to Figure 5. Circular plots of firing distribution of individual layer II (green, n=18) and layer III (brown, n=10) pyramidal cells.

Table S1, related to Figure 2 and 3. Electrophysiological characteristics of functional groups of FS cells recorded and labeled in drug-free conditions. Spike amp: spike amplitude; Spike width: spike half-amplitude width; DV: dorsal-ventral coordinates; ML: medial-lateral coordinates; AP: anterior-posterior coordinates; PV: parvalbumin.

Cell group and cell code	Spike amplitude[a] (mV)	Spike width[b] (ms)	Recording duration[c] (min:sec)	Firing rate (Hz) in SWS[d]	Number of spindle troughs		Mean vector direction[g] (degree)	Mean vector lengths[h]	Anatomical coordinates (mm)			PV	Number of peaks in spike timing (trough ± 6.25 ms)	Number of peaks in spike timing (trough ± 25 ms)
					all [e]	without spikes** [f]			DV	ML	AP			
Spindle ripple cells														
rat78d1t1c1	1.4	0.135	34:30	26.89	1138	241	9.31	0.284	160	0.83	3.24	+	3	3
rat95d3t2c1	1.0	0.165	42:00	29.45	1657	599	13.28	0.32	325	2.37	-4.08		3	3
rat121d1t3c1	2.2	0.18	13:16	27.5	1124	238	-17.83	0.335	250	2.5	-3.96		3	3
Mean	1.53	0.160	29:55	27.95	1306.3	359.3	1.06	0.31						
SD	0.61	0.023	14:54	1.34	303.8	207.6	14.05	0.03						
Median	1.40	0.165	34:30	27.50	1138.0	241.0	9.31	0.32						
Spindle high gamma cells														
rat119d1t1c2	1.1	0.12	22:30	34.21	701	95	6.35	0.3441	745	2.57	-3.76	+	2	2
rat72d1t1c1	0.25	*	14:00	32.7	646	102	-11.95	0.19	200	2.2	-4.0		2	2
rat131d2t1c2	0.85	0.14	15:00	21.2	580	67	-4.84	0.5	476	2.9	-3.72	+	2	2
rat35d1t1c4b	1.2	0.165	24:00	33.5	2151	246	-12.43	0.379	300	2.2	-2.72		2	2
Mean	0.85	0.142	18:52	30.40	1019.5	127.5	-5.12	0.35						
SD	0.43	0.073	05:06	6.17	756.0	80.4	7.25	0.13						
Median	0.98	0.140	18:45	33.10	673.5	98.5	-8.40	0.36						
Simple spindle cells														
rat36d1t3c2	1.5	0.15	07:40	21.95	263	28	9.31	0.554	450	2.0	4.0		1	1
rat133d1t1c1	1.34	0.245	43:00	9	1650	924	-3.22	0.64	364	2.0	-3.1	+	1	1
rat139d1t2c4	1.7	0.12	14:00	28.46	769	156	-16.67	0.26	594	2.47	-2.85	+	1	1
rat38d1t2c1	1	0.13	10:48	16.67	497	272	-7.2	0.33	350	3.0	-3.48		1	1
rat68d2t2c1	1.1	0.135	23:00	33.4	1684	225	24.92	0.34	200	2.4	-3.48		1	1
rat69d2t1c2	0.75	0.16	06:30	31.81	385	52	-12.19	0.424	230	2.65	-3.96		1	1
rat79d2t2c2	0.3	*	40:00	27	903	166	-9.45	0.725	400	2.0	4.0		1	1
Mean	1.10	0.157	20:43	24.04	878.7	369.3	-2.28	0.47						
SD	0.47	0.072	15:13	8.77	580.9	342.4	11.88	0.17						
Median	1.10	0.143	14:00	27.00	769.0	156.0	-7.20	0.42						
Spindle descending phase cells														
rat74d3t2c1	0.35	*	16:00	28.7	502	47	-47.8	0.6213	500	2.35	-3.27		0	1
rat157d1t1c3	0.6	0.13	23:26	48.34	2512	667	-42.37	0.265	580	2.1	-2.94		0	1
rat81d1t1c1	1.75	0.175	32:10	37.7	720	89	-22.57	0.32	306	2.4	-3.28	+	0	1
rat87d2t3c4	2.3	0.13	30:00	24.3	1502	175	-41.52	0.6	487	2.3	-3.4	+	0	1
Mean	1.25	0.145	25:24	34.76	1309.0	244.5	-40.49	0.45						
SD	0.93	0.076	07:17	10.63	909.7	286.7	8.70	0.19						
Median	1.18	0.130	26:43	33.20	1111.0	132.0	-41.95	0.46						

Complex (ungrouped) cells														
rat51d1t1c1	1.1	0.135	17:50	31	833	152	-2.5	0.13	222	2.3	-2.7	+	2	3
rat101d2t2c1	1.4	0.175	21:00	34.2	1384	164	-6.47	0.36	162	2.95	-3.6		2	3
rat126d1t2c2	1	0.16	25:00	24	1505	369	-4.93	0.31	293	2.4	-2.82		2	3
Mean	1.17	0.157	21:17	29.73	1240.7	228.3	-5.23	0.27						
SD	0.21	0.020	03:35	5.22	358.2	122.0	1.39	0.12						
Median	1.10	0.160	21:00	31.00	1384.0	164.0	-4.93	0.31						

* Spike half widths are not indicated due to low amplitude of the spikes.
** Time window without spikes was ± 25 ms around the troughs of spindle cycles.
The mean vector direction (MVD) of spindle descending phase cells significantly ([g] $p < 0.047$, random permutation test) preceded the MVD measured in either functional cell groups. No other parameters were significantly different between cell groups (Kruskal-Wallis test, [a] $p = 0.707$; [b] $p = 0.736$; [c] $p = 0.679$; [d] $p = 0.404$; [e] $p = 0.651$; [f] $p = 0.560$; [h] $p = 0.421$).
